# Effects of anti-PD-1 immunotherapy on tumor regression: insights from a patient-derived xenograft model

**DOI:** 10.1038/s41598-020-63796-w

**Published:** 2020-04-27

**Authors:** Asunción Martín-Ruiz, Carmen Fiuza-Luces, Esther Martínez-Martínez, Clemente F. Arias, Lourdes Gutiérrez, Manuel Ramírez, Paloma Martín-Acosta, Maria José Coronado, Alejandro Lucia, Mariano Provencio

**Affiliations:** 1 Medical Oncology Department, Hospital Universitario Puerta de Hierro Majadahonda, Madrid, Spain; 2 Faculty of Sports Sciences, European University, Madrid, Spain; 3 Research Institute of the Hospital 12 de Octubre (imas12), Madrid, Spain; 4 Department of Biology, University of Fribourg, Fribourg, Switzerland; 5 Departament of Applied Mathematics, Universidad Complutense de Madrid, Madrid, Spain; 6 Oncohematology Department, Children’s Hospital Niño Jesús, Madrid, Spain; 7 Pathology Department, Hospital Universitario Puerta de Hierro Majadahonda, Madrid, Spain; 8 Confocal Microscopy Core Facility, Hospital Universitario Puerta de Hierro Majadahonda, Madrid, Spain

**Keywords:** Cancer, Cancer microenvironment, Cancer models, Cancer therapy, Lung cancer, Tumour immunology

## Abstract

Immunotherapies, such as checkpoint blockade of programmed cell death protein-1 (PD-1), have resulted in unprecedented improvements in survival for patients with lung cancer. Nonetheless, not all patients benefit equally and many issues remain unresolved, including the mechanisms of action and the possible effector function of immune cells from non-lymphoid lineages. The purpose of this study was to investigate whether anti-PD-1 immunotherapy acts on malignant tumor cells through mechanisms beyond those related to T lymphocyte involvement. We used a murine patient-derived xenograft (PDX) model of early-stage non–small cell lung carcinoma (NSCLC) devoid of host lymphoid cells, and studied the tumor and immune non-lymphoid responses to immunotherapy with anti-PD-1 alone or in combination with standard chemotherapy (cisplatin). An antitumor effect was observed in animals that received anti-PD-1 treatment, alone or in combination with cisplatin, likely due to a mechanism independent of T lymphocytes. Indeed, anti-PD-1 treatment induced myeloid cell mobilization to the tumor concomitant with the production of exudates compatible with an acute inflammatory reaction mediated by murine polymorphonuclear leukocytes, specifically neutrophils. Thus, while keeping in mind that more research is needed to corroborate our findings, we report preliminary evidence for a previously undescribed immunotherapy mechanism in this model, suggesting a potential cytotoxic action of neutrophils as PD-1 inhibitor effector cells responsible for tumor regression by necrotic extension.

## Introduction

Cancer immunotherapy, in particular antibody-based immune checkpoint blockers, represents a revolution in cancer treatment, generating unprecedented results in terms of overall and progression-free survival^1,2^. Several of the most studied immune checkpoint targets, such as programmed cell death protein-1/programmed death ligand-1 (PD-1/PD-L1 axis), are involved in escape mechanisms from immunosurveillance^2–4^. Accordingly, anti-PD-1 therapies (e.g., nivolumab) are based on improving the anti-tumor immune response against cancer cells, principally by stimulating the infiltrating cytotoxic T lymphocytes (CD8+) in the tumor microenvironment^5–7^. These therapies block the immunoinhibitory receptor PD-1, which is normally expressed on the surface of activated T cells, regulatory T cells, B cells and natural killer (NK) cells^3,6,7^.

However, in addition to low objective response rate for some tumors, notably for non–small cell lung carcinoma (NSCLC) and nivolumab^8,9^, there is a wide inter-individual variability of response to anti-PD-1 therapy, which complicates the task of reliably identifying responders and non-responders. Moreover, there are no robust markers to predict which patients are most likely to benefit from this therapy^10^. Indeed, the determination of tumoral PD-L1 expression by immunohistochemistry (IHC), as well as the quantification of lymphocytes and tumor-infiltrating lymphocytes (TILs) are being questioned as markers of predictive value of response^7,11^. Furthermore, tumor PD-1 expression is not considered a useful predictor of the response to anti-PD-1 therapy, and in fact in patients with NSCLC – which accounts for the vast majority of lung malignancies – only tumoral PD-L1 is determined (with results being considered positive if expression levels >1%)^12–14^. In fact, individuals with negative results for PD-L1 expression have a treatment response comparable to those showing positive expression^15,16^. There is therefore a need to identify new mechanisms related to specific aspects of the tumor-immune system interaction and independent from lymphoid cells.

The purpose of this study was to investigate whether anti-PD-1 immunotherapy acts on malignant tumor cells through mechanisms other than the classically advocated T lymphocyte involvement. To this end, we used a murine patient-derived xenograft (PDX) model of squamous NSCLC devoid of host lymphoid cells, and compared the tumor and immune non-lymphoid responses to immunotherapy with anti-PD-1 alone or in combination with standard chemotherapy.

## Material and Methods

Ethical approval for the collection and use of patient tumor tissue was granted by the Ethics Committee of the *Hospital Universitario Puerta de Hierro* [HUPH] (Madrid, Spain; approval number: PI/144-14), and the study was conducted in accordance with the Declaration of Helsinki during May 2014–October 2018. Eligibility criteria were the following: new diagnosis of a primary lung tumor in patients with NSCLC, not having received previous therapy other than surgery, provision of a sufficient quantity of tumor volume to donate a section for research purposes, and no history of infectious diseases. All participants provided written informed consent.

All animal experimental protocols were approved by the institutional review committee (HUPH, approval number: PROEX 163/14) and were conducted in accordance with European (European convention ETS 123) and Spanish (32/2007 and R.D. 1201/2005) laws on animal protection in scientific research. NOD-SCID gamma (NSG) mice were housed in the animal facility of HUPH, in laminar airflow cabinets under specific pathogen-free conditions and on a 12-hour light/12-hour dark cycle with *ad libitum* access to food and water.

### Study design

Details on the establishment of the human squamous NSCLC PDX model used for this study and on the preliminary test of response to anti-PD-1 therapy are shown in text file S1 in Supplementary Material.

#### Pharmacological intervention

**Figure 1:**
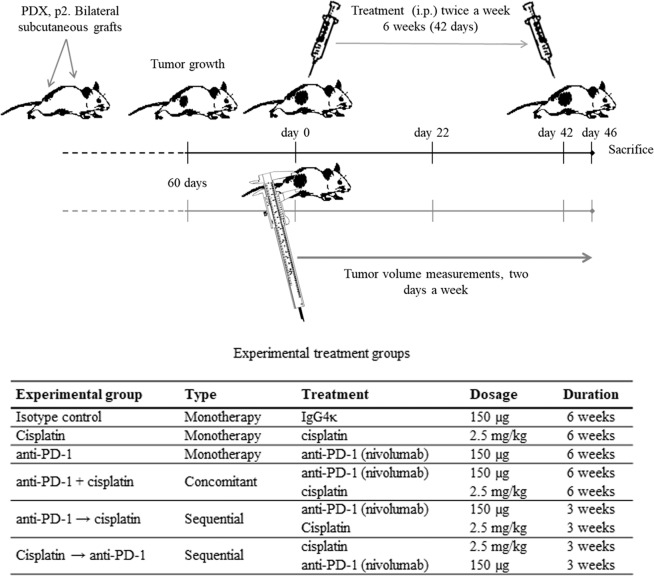
Study design with NOD-SCID gamma (NSG) mice. Abbreviation: PDX, patient-derived xenograft.

Transplanted p2 mice were monitored and their bilateral tumor volumes were measured (Fig. 1). When the tumors reached the appropriate size (~100 mm^3^), mice were randomly assigned to the following experimental groups (4 mice [and thus 8 tumors]/group) and the treatment was initiated (twice weekly for 6 consecutive weeks – from day 0 to 42): isotype control, cisplatin (monotherapy), anti-PD-1 (monotherapy), cisplatin + anti-PD-1 (concomitant group), sequential treatment with cisplatin and anti-PD-1 (cisplatin → anti-PD-1) or *vice versa* (anti-PD-1 → cisplatin) (Fig. 1). Finally, 2 days after the last treatment administration (day 46), blood samples were taken from the tail vein and collected in EDTA tubes and the mice were sacrificed as described above. The tumor tissue was harvested and divided into pieces, which were freshly conserved and embedded in Tissue-Tek OCT compound (Sakura Finetechnical Co., Ltd.; Tokyo, Japan) or in paraffin for subsequent analysis.

#### Outcomes

##### Tumor stability analysis during consecutive passaging

To verify that the histopathological features of the xenograft tumors were stable and similar to patients´ tumors during the subsequent passages, we performed several analyses. Tumors were fixed (10% formalin), embedded in paraffin (PANREAC Applichem; Darmstadt, Germany), sectioned at 4 µm, immunostained using the Dako Cytomation autostainer (Dako Diagnostics; Barcelona, Spain) or the Leica Bond-Max System (Leica Microsystems; Wetzlar, Germany), and counterstained with hematoxylin and eosin (H&E). Histopathological analysis was then carried out to assess the type and histologic tumor subtype, the degree of cellular differentiation and the tumor infiltration. The differential diagnosis of NSCLC was made on paraffin sections with an immunophenotype panel (Table S1 in Supplementary Material, IHC markers shaded in light gray). The immunophenotype analysis was compared with the expression pattern obtained from the respective patient. The tumor expression of hCD45, hPD-1 and hPD-L1 was also determined by IHC (Table S1 in Supplementary Material, markers shaded in dark gray). In addition, to confirm that the tumor cells were human, we analyzed the presence of *Alu sequences* on paraffin-embedded tissue using the Alu Positive Control Probe II (Ventana Medical Systems Inc.; Roche Diagnostics; Mannheim, Germany) for the automated Ventana BenchMark Instrument.

##### Tumor volume and tumor growth rate

Tumor volumes were measured twice weekly throughout the treatment period and at the day of sacrifice using a caliper, as previously mentioned. We assessed the response to therapy in terms of tumor volume using the rate of tumor growth, which refers to the percentage of tumor growth with respect to the volume at the beginning of the therapy, calculated by the formula (TVdx/TVd0) × 100, where TVdx refers to the tumor volume measured on a specific day and TVd0 is the tumor volume at the beginning of treatment administration (set at 100%). Tumor growth curves were generated for each mouse.

##### Necrotic index

To assess tumor regression in response to therapy we calculated the percentage of necrotic areas in paraffin-embedded sections stained with H&E. Four sections of each tumor were scanned and the necrotic areas were quantified using CaseViewer software (3DHISTECH Ltd., Budapest, Hungary) with the formula % necrosis area = (Σ necrosis area/total tumoral mass area) × 100.

##### Cell identification in tumor-derived fluids (*in vivo*)

We observed some tumor-derived fluids during tumor harvesting *in vivo* and tumor fragmentation *ex vivo* (see Results). The fluids were collected, fixed in 95% ethanol for 15 minutes, and stained using the standard trichrome Papanicolaou method. Fluids were analyzed by liquid cytology using the ThinPrep Pap Test (Cytyc Corporation; Boxborough, MA, USA).

##### Leukocyte identification in peripheral blood (*in vivo*) and in tumor stroma (*ex vivo*)

Complete peripheral blood (conserved in EDTA tubes) and cell suspensions from tumor tissue were processed by flow cytometry using a FACSCanto II instrument and FACSDiva software v6.1.2 (BD Biosciences; Franklin Lakes, NJ, USA). Individual fresh tumors (~0.1 g) were homogenized, digested with 1 mg/ml collagenase D (Roche Diagnostics; Mannheim, Germany) for 24 hours and filtered through a 40-μm nylon mesh cell strainer (BD Bioscience). Flow cytometry analyses were performed using fluorochrome-conjugated monoclonal antibodies for human and mouse antigens (Table S2 in Supplementary Material). Dead cells were excluded by 7-aminoactinomycin D staining. Specific antibodies for human and mouse immune cells were used for classification of the different cellular populations (the combination of antibodies are shown in Table S3 in Supplementary Material).

##### Identification of tumor-infiltrating cells

Cryostat sections of tumors preserved in OCT were fixed with 10% formalin, washed with phosphate-buffered saline (PBS) (3 × 5 minutes with shaking) and incubated in 50 mM NH_4_Cl (5 minutes). Thereafter, the samples were permeabilized with 0.2% Triton X-100 for 10 minutes and subsequently blocked with 5% bovine serum albumin (BSA) to reduce non-specific protein binding. The samples were incubated with the primary antibodies (non-shaded markers, Table S1 in Supplementary Material) anti-myeloperoxidase (MPO [1/25]), anti-nitrotyrosine (1/25) or anti-PD-1 (1/200) overnight at 4 °C, shaking, washed 3 times with PBS and then incubated with secondary antibodies diluted 1/500 in 1% BSA for 45 minutes at room temperature (Table S4 in Supplementary Material). Nuclei were stained with TO-PRO-3 (Thermo Fisher Scientific; Waltham, MA, USA). Finally, slides were washed 3 times in PBS and the coverslips were mounted in PBS/glycerol. Images were obtained using a confocal ultra-spectral microscope (Leica TCS-SP5-AOBS-UV, Leica-Microsystems; Wetzlar, Germany), with a 20× objective and 0.4 numerical aperture.

A similar protocol was followed for double immunofluorescence staining with secondary antibodies for MPO/nitrotyrosine and for MPO/PD-1 (Table S4 in Supplementary Material).

### Experiments for neutrophil phenotype characterization

#### Isolation of neutrophils from peripheral blood of NOD-SCID gamma mice

Peripheral blood of NOD-SCID gamma mice was drawn from the subclavian vein after an intraperitoneal injection of a sublethal dose of anaesthesia (Avertin 0.2%, 0.16 ml/g [Sigma-Aldrich; St. Louis, MO, USA]) and collected in EDTA BD microtainer tubes (BD Biosciences; Franklin Lakes, NJ, USA). All mice were killed by cervical dislocation. After removing red blood cells using Quicklysis solution (Cytognos SL; Salamanca, Spain), neutrophils were isolated and purified following the Neutrophil Isolation Kit protocol (Miltenyi Biotec; Bergisch Gladbach, Germany [reference # 130-097-658])^17–19^. The degree of the neutrophil purity achieved (CD11b + Ly6G+ cells; Table S3 in Supplementary Material) was assessed using standard sorting procedures by flow cytometry.

#### Neutrophil nuclear morphology after exposure to anti-PD-1

Nuclear morphology was studied in neutrophils from tumor exudates treated with anti-PD-1 using liquid citology (ThinPrep Pap Test), as well as in the immunofluorescence images of isolated neutrophils exposed to anti-PD-1, applying a negative photo filter with the invert option in Adobe Photoshop CC (version 2017.0.0; Adobe Systems Inc., San Jose, CA, USA).

#### Immunofluorescence experiments after exposure to anti-PD-1

A suspension of isolated and purified neutrophils was exposed to either anti-PD-1 (nivolumab) or isotype control, both at 50 µg/ml for 1 hour. Subsequently, neutrophils were fixed with 4% paraformaldehyde and incubated with fluorescein isothiocyanate (FITC)-conjugated anti-hIgG for 45 minutes (Table S4 in Supplementary Material) to detect the binding of anti-PD-1, through its antigen-binding fragment (Fab) variable region, to the PD-1 receptor of the neutrophil membrane cell surface. For a second immunodetection, the samples were incubated with a recombinant human PD-1 protein (active)-phycoerythrin (PE) ligand (Abcam; Cambridge, UK [reference # ab246145]) applied for 1 hour and using a concentration of 1/500 diluted in PBS (Table S4 in Supplementary Material). This second staining was used to detect if anti-PD-1 had bound, through its fragment crystallizable (Fc) region, to Fcγ receptors (FcγR) in the neutrophil cell membrane surface, thereby leaving the ligand binding site free. After washing the cells with PBS (twice), they were mounted with a mixture of PBS:glycerol. These experiments were also performed exposing neutrophils for 1 hour to an isotype control, human immunoglobulin G4 (hIgG4).

Images were collected with a TCS SP5 confocal microscope (Leica Microsystems; Wetzlar, Germany) using a 63× HCX PL APO (1.4 numerical aperture) and processed with the ASF Leica software (Leica Mycrosystems) as detailed elsewhere^20^. Excitation and emission parameters: 488 nm/500–540 nm and 546 nm/557–572 nm, for FITC−conjugated anti-hIgG and for PD-1−PE, respectively.

### Statistical analysis

Data are presented as mean ± SEM for all figure panels in which error bars are shown. Tumor growth curves were compared between treatment groups with the non-parametric Kruskal-Wallis test and statistical significance was set at 0.05. The extent of necrosis was expressed as the percentage of necrotic tissue and between-group analysis was performed using the Kruskal-Wallis test. Analysis was performed using IBM SPSS 22.0 software (SPSS, Inc.; Chicago, IL, USA). All graphics were made with GraphPad Prism 6, version 6.01 software (GraphPad Software; San Diego, CA, USA).

## Results

### Tumor stability during consecutive passaging in mice

Fresh tumor tissues were obtained from 17 patients to generate the PDX models (clinical patient data is shown in Table S5 in Supplementary Material). Histopathological characteristics of the passaged NSCLC PDX tumors remained stable in morphology, histological type and tumor cell differentiation grade (Fig. S1 in Supplementary Material).

### Sensitivity response test to anti-PD-1 therapy

As shown in the flow diagram (Fig. S2 in Supplementary Material), a total of 10 NSCLC PDX lines were established. Of those, PDX lines derived from squamous cell lung cancer were chosen, and tumors with a known driver mutation were excluded because of the effect the mutation may have on the treatment outcomes. We also excluded PDX lines that showed tumor degeneration with keratinized phenotypes, because of the difficulty of adequately assessing the tumor sensitivity to therapy caused by the massive production of keratin in these cases. Some PDX lines included mice with atypical Epstein-Barr virus-associated human B cell lymphomas and, for this reason, they were also not used. Finally, only one PDX line showed a clear response in view of changes in tumor volumes in the grafts, and this was selected as a model to study the effect of the immunotherapy treatment in the absence of lymphocytes. The tumor sample of this PDX line (PDX4) came from a 79-year-old Caucasian male patient who was submitted to a right upper lobectomy with complete resection (Table S5 in Supplementary Material). According to WHO criteria for histological classification and staging, the tumor was a basaloid infiltrating and poorly differentiated squamous cell lung carcinoma, and the patient was staged as IIA (pT2a N1 L1 M0).

We also present results from an additional PDX line – PDX6, which was a non-responder to immunotherapy – derived from a patient whose characteristics are shown in Table S5 in Supplementary Material.

### Tumor volume and tumor growth rate

**Figure 2:**
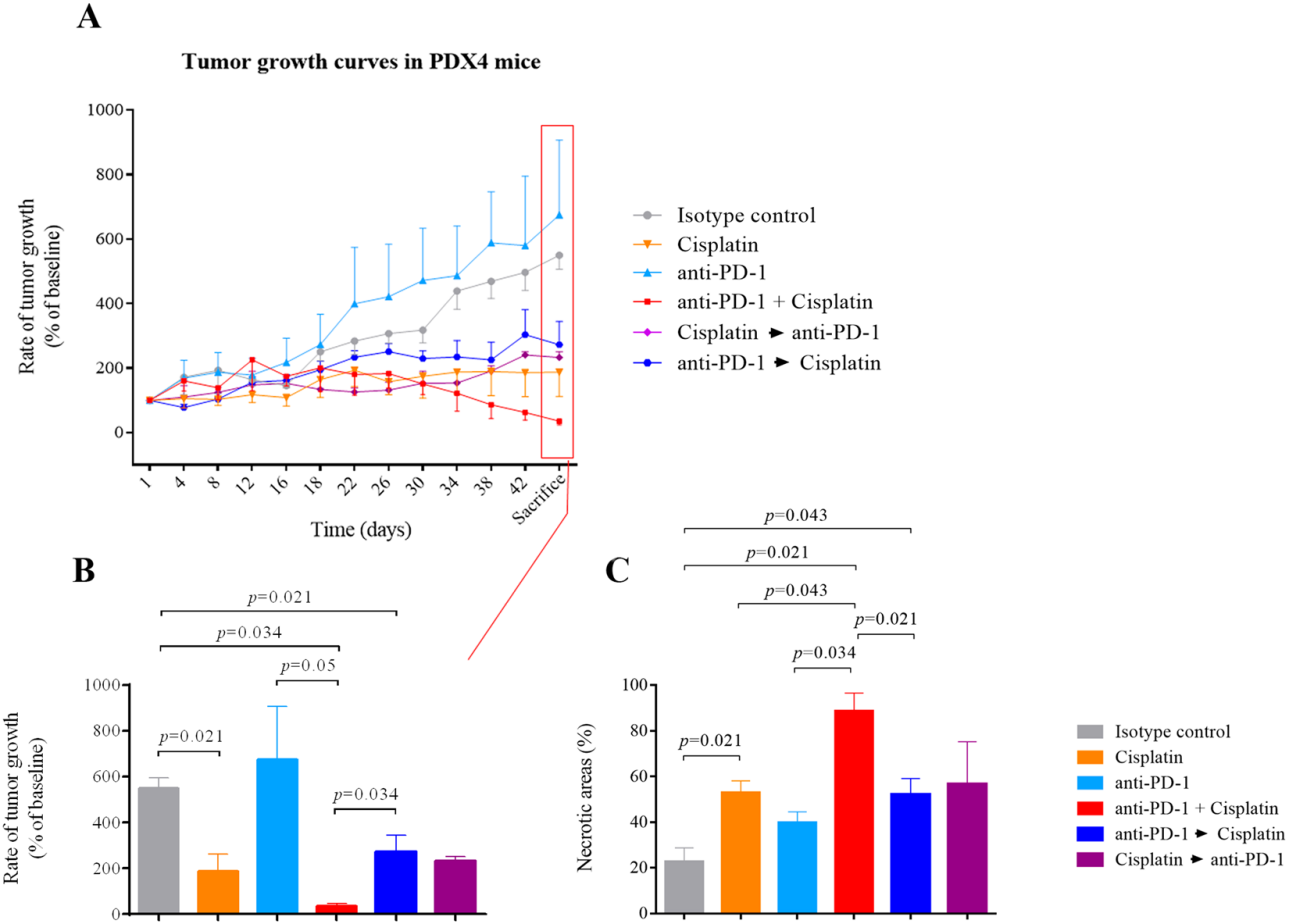
Effect of different treatments on tumor (non–small cell lung carcinoma) size and on tumor necrosis in patient-derived xenograft PDX4 in NSG mice. (**A**) Tumor growth curves are expressed as a percentage of the change of initial tumor volume, which was considered as 100%. Each point represents a measurement day given as the mean ± SEM of the tumor volume of the mice of each group (2 tumors per mice, *i.e*., n = 8 tumors per group). **(B)** Mice were sacrificed after six weeks and the tumor volume was measured. Results are shown as mean ± SEM of the tumor volume of the mice of each group (n = 8 tumors per group). Statistical significance is indicated (p ≤ 0.05) and was analyzed by the Kruskal-Wallis non-parametric test. Post hoc between-group comparisons were made with the Mann-Whitney test. **(C)** The necrosis index is expressed as a percentage of tumor necrotic areas (n = 6–8 tumors per group). Values represent the percentage (mean ± SEM). Statistical significance is indicated (p ≤ 0.05) and was analyzed by the Kruskal-Wallis non-parametric test. Post hoc between-group comparisons were made with the Mann-Whitney test.

We analyzed the tumor growth rates in all experimental groups of PDX4 from the beginning to the end of the study (Fig. 2A). Briefly, in the anti-PD-1 group, we found a progressive increase in the tumor volume over time as compared with the other treatment groups (p = 0.011 for the group effect), and a similar pattern was observed in the isotype control group, showing the natural evolution of the disease. As anticipated, growth rates in the cisplatin-administered group were significantly lower than in the isotype control group. However, the lowest tumor growth rate was found in the cisplatin + anti-PD-1 (concomitant) group. With regard to the sequential treatments (anti-PD-1 → cisplatin and *vice versa*), the tumor growth rates followed a specular path, with an increase in tumor volumes during the period of anti-PD-1 administration.

Considering the tumor volume at sacrifice, we found the highest volume in both the anti-PD-1 and isotype control groups and the lowest volume in the concomitant treatment group (Fig. 2B, where all the numerous post hoc between-group differences are shown), which was even lower than that measured in the cisplatin group. Tumor volumes between the sequential treatment groups were not significantly different; however, the final tumor volume in the anti-PD-1 → cisplatin group was lower than that in the isotype group and higher than in the concomitant group (Fig. 2B).

However, as shown in Figs. S3A,B in Supplementary Material, no changes were found in the tumor volume and growth rate of PDX6 mice with regard to anti-PD-1 monotherapy.

### Necrosis

Given these results, in particular the increase in tumor volume of PDX4 after anti-PD-1 administration, we next evaluated the extent of tumor necrosis for the different treatments. After calculating the necrosis index in the different experimental groups (expressed as the percentage of necrotic areas by total surface estimated by H&E staining), we found a significant group effect (p = 0.014) with the highest values observed in the concomitant treatment group (anti-PD-1 + cisplatin) and the lowest values in the isotype control group (corresponding to normal conditions of squamous histology) (Fig. 2C).

Concomitant treatment increased tumor necrosis and significantly decreased the viable component of the tumor (Fig. 2C, where all the numerous post hoc between-group differences are shown, and Fig. S4A in Supplementary Material), achieving the greatest tumor regression, which was superior to that achieved by anti-PD-1 → cisplatin sequential treatment. However, the aforementioned sequential treatment group presented a significantly higher necrotic index than the isotype control group (p = 0.043) but statistical significance was not reached when compared to the cisplatin → anti-PD-1 group. There was a trend for an increase in the necrotic index in the anti-PD-1 group as compared with the isotype control group (p = 0.068). Overall, the percentage of necrotic areas tended to be higher in all experimental groups that included the anti-PD-1 and/or cisplatin treatment as compared with the isotype control group. By contrast, in the PDX6 model (Fig. S3C in Supplementary Material) we only found a significant difference (p = 0.043) in the percentage of necrotic areas after anti-PD-1 → cisplatin sequential treatment compared with the isotype control group, with no differences between the latter and the remainder of groups.

### Identification of cells in tumor-derived fluids

**Figure 3:**
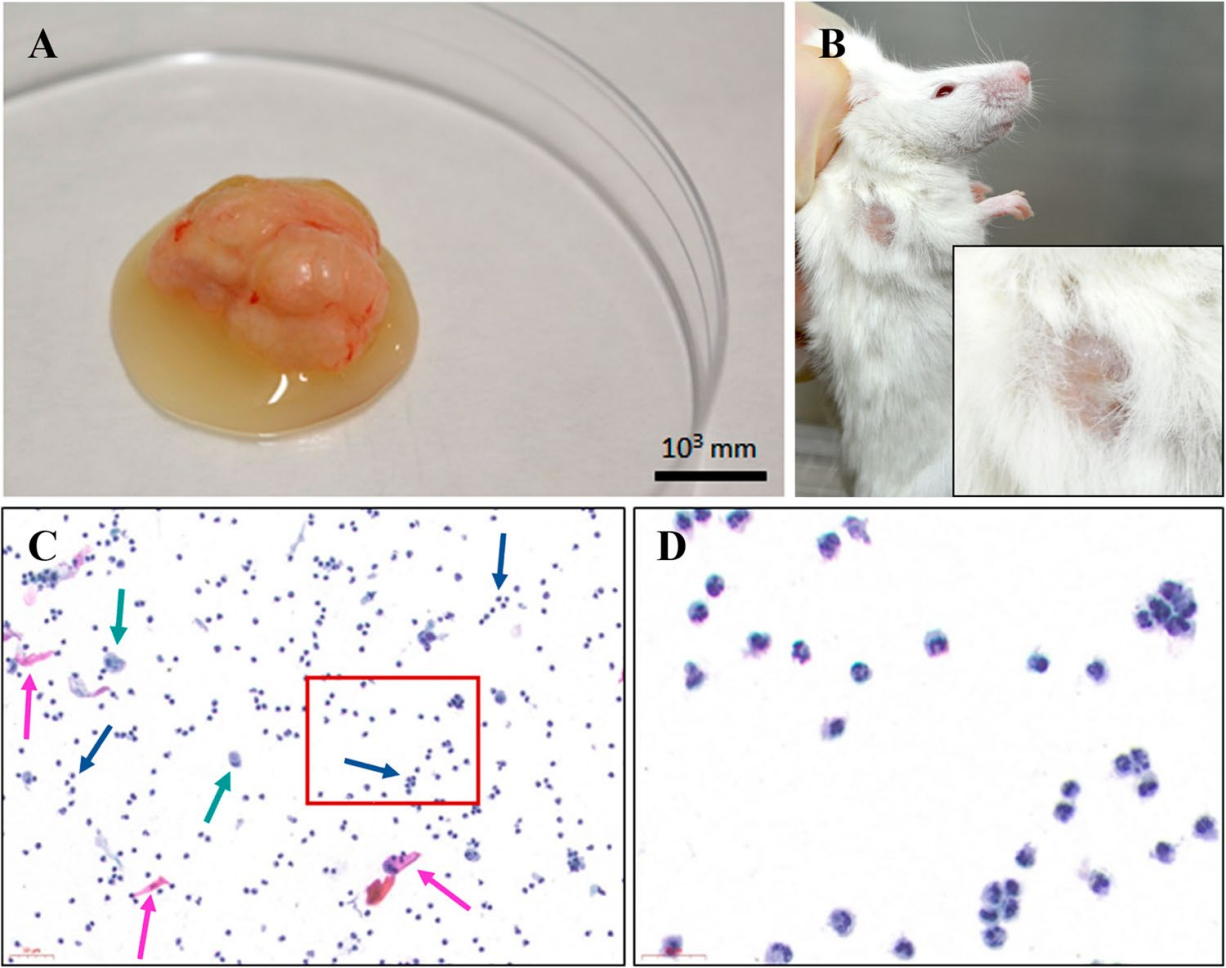
Anti-PD-1 administration is associated with an acute inflammatory reaction. (**A**) Shown is a representative patient-derived xenograft PDX4 tumor treated with anti-PD-1 monotherapy, characterized by the formation of a fluid exudate. **(B)** Shown is a representative example of a mouse in the anti-PD-1 + cisplatin (concomitant) treatment group, presenting ‘wet hair’ caused by the exudate of the subcutaneous tumor – the ‘wet hair’ image is also zoomed. **(C)** Liquid cytology (*ThinPrep, Cytyc Corporation; Boxborough, MA, USA*) of the exudate showing dead epithelial cells (pink arrows) and cell debris from necrotic tissue and also inflammatory cells such as polymorphonuclear cells (blue arrows), and macrophages (green arrows), magnification ×20. **(D)** Liquid cytology of polymorphonuclear cells, magnification ×75.

Tumors treated with anti-PD-1 contained a fluid with a serous appearance at the time of tumor removal (Fig. 3A), and this was also evident through a break in the skin during *in vivo* measurements (Fig. 3B). Because the fluid was only observed in the anti-PD-1 group (in both monotherapy and in combination), it was unlikely that this was due to necrosis associated with the tumor itself. Indeed, tumors from the anti-PD-1 + cisplatin group exuded fluids prominently (Fig. 3B), despite presenting the smallest tumor volume among the experimental groups. Moreover, although the tumors treated with anti-PD-1 in monotherapy were the largest at the end of the experiment (Fig. 2A), many of them were liquified (Fig. 3A) and had large internal cavities. After two expert pathologists had analyzed all the samples with liquid cytology, we determined that this fluid corresponded to a cellular exudate compatible with an acute inflammatory reaction, as reflected by the presence of debris of dead squamous cells and a large number of myeloid cells or polymorphonuclear (PMN) leukocytes specifically neutrophils, as well as debris of dead squamous cells (Figs. 3C,D).

### Leukocyte identification in peripheral blood and in tumor stroma

**Figure 4:**
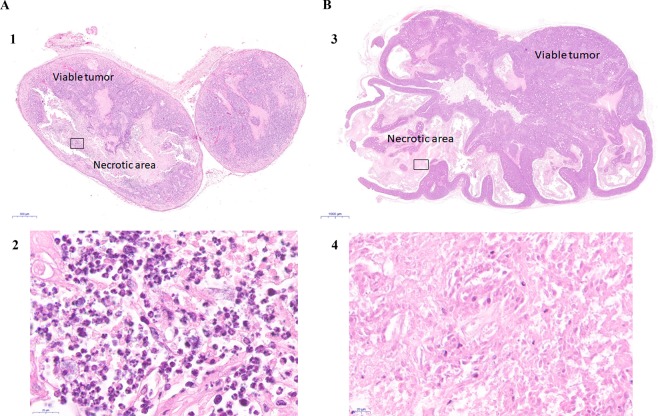
Tumor PDX histologies and inflammatory response associated with tumor regression in response to anti-PD-1 treatments in ‘responder’ (PDX4, **A**) and ‘non-responder’ tumors (PDX6, **B**). (**1**) PDX4 tumor treated with anti-PD-1 stained with H&E (×2). **(2)** Amplified image of the area inside the square in (1), where necrotic areas with inflammatory cell – mainly neutrophils – infiltrates can be seen (x100). **(3)** PDX6 tumor treated with anti-PD-1 stained with H&E, where necrotic areas and viable tumor areas are indicated (×2). **(4)** Amplified image of the area inside the square in (3), where necrotic areas without inflammatory infiltrates (no neutrophils) can be seen (x100).

We tested for the presence of human leukocytes (hCD45+) in peripheral blood in the study groups by flow cytometry, which was negative in all cases (Fig. S5A in Supplementary Material). However, consistent with our observations in the tumor/cellular exudates in the anti-PD-1 groups, we observed the accumulation of inflammatory PMN cells coinciding with the extensive necrotic areas in tumor sections stained with H&E (Figs. 4A, S4B,C in Supplementary Material). By contrast, no such phenomenon was observed in the tumors of the non-responder (PDX6) mouse line after anti-PD-1 treatment (Figs. 4B, S4D,E in Supplementary Material). In the latter, we found no inflammatory infiltrate in necrotic areas, which solely included debris of necrotized tissue and dead epithelial (carcinoma) cells.

To ascertain whether aforementioned PMN cells found in tumor exudates in were possible candidates for anti-PD-1 therapy effector cells, we first determined whether they were lymphocytes (*a priori*, the immunotherapy target cells). However, after several different analyses, we determined that these cells were neutrophils, as detailed below.

Using a human-specific Alu-sequence, we determined that the xenograft stroma included both human and mouse stromal components, (Figs. S5D,E in Supplementary Material). Further, IHC analysis using a panel of antibodies ruled out the presence of specific lymphocyte subpopulations in tumors, treated or not with anti-PD-1 (Fig. S5F in Supplementary Material).

We next utilized flow cytometry panels to examine the infiltrated leukocyte component from tumor homogenates as well as from fluids collected from some tumors treated with anti-PD-1. In both cases, analysis showed 0% human (hCD45+) and 100% murine (mCD45.1+) leukocyte components, and the results were very similar independently of the experimental group (Figs. S5B,C in Supplementary Material). Thus, we considered that the lineage present in the samples was the host myeloid.

### Neutrophil nuclear morphology

Liquid cytology and subsequent immunofluorescence analysis performed in tumor exudates from mice treated with anti-PD-1 allowed us to study if the morphology of PMN after treatment exposure corresponded to that of neutrophils. As shown in Fig. S6 in Supplementary Material, a multilobed and hypersegmented nucleus that is characteristic of neutrophils, and specifically of neutrophils with the N1 (i.e., anti-tumoral) phenotype, was recognized in the analyzed images. Confocal microscopy images corroborated this finding.

### Neutrophil activation in tumors

**Figure 5:**
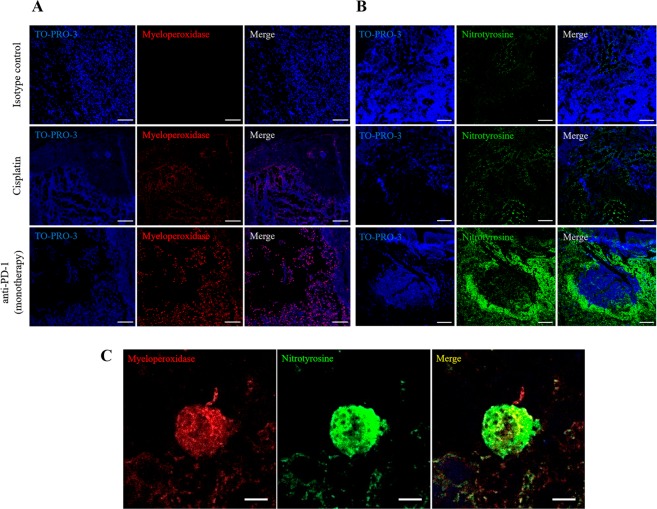
Recruitment of neutrophils and nitrotyrosine formation with anti-PD-1 treatment. (**A**) Representative confocal microscopy images of myeloperoxidase (red) staining of neutrophils in necrotic areas in tumors from the isotype control group, and from the experimental groups treated with cisplatin or anti-PD-1 monotherapy. Nuclei were stained with TO-PRO-3 (blue). Right panels show merged images. Scale bars, 100 µm. **(B)** Nitrotyrosine labeling in necrotic areas from the anti-PD-1 treatment group. Representative images of nitrotyrosine (green) localization by confocal microscopy in each study group. Nuclei were stained with TO-PRO-3 (blue). Right panels show merged images. Scale bars, 100 µm. **(C)** Detection of myeloperoxidase and nitrotyrosine in infiltrated tumor cells of mice treated with anti-PD-1. Representative confocal microscopy images of expression and localization of myeloperoxidase (red) and nitrotyrosine (green) in tumors from the control group and from tumors treated with anti-PD-1 monotherapy. Right panel shows merged images of co-localization (yellow). Scale bars, 5 µm.

Given the identification of neutrophils as the potential anti-PD-1 therapy effector cells, we evaluated elements of the neutrophil oxidant pathway, specifically MPO and nitric oxide (NO), in tumors from the cisplatin and anti-PD-1 (monotherapy) groups and from the isotype control group, as a possible mechanism for the cytotoxic action of neutrophils during immunotherapy treatments. As shown in Fig. 5A, neutrophil infiltration, measured by MPO staining, coincided with the necrotic areas from tumors treated with anti-PD-1, which was not the case with the isotype control group. Also, necrosis in the cisplatin group was accompanied by tissue degradation and structural disruption (see TO-PRO 3 staining in cisplatin treatment, Figs. 5A,B). We assessed the presence of NO indirectly by evaluating nitrotyrosine modification. Whereas the expression of nitrotyrosine was essentially absent in the isotype control group, some labeling could be detected in the groups treated with cisplatin (Fig. 5B). By contrast, there was a very strong labeling of nitrotyrosine in the anti-PD-1 group (Fig. 5B), which was particularly evident around the necrotic areas where presumably NO is released.

### Immunofluorescence experiments in tumors: Identification of tumor-infiltrating cells

Double immunofluorescence experiments revealed the co-localization of MPO and nitrotyrosine in neutrophils from tumors treated with anti-PD-1 (Figs. 5C and S7A in Supplementary Material), but not in the isotype control or the cisplatin groups (Fig. S7B in Supplementary Material), which would suggest that NO production in neutrophils is a response to anti-PD-1 treatment. In accord with this, nitrotyrosine and MPO co-localization was detected in the sequential anti-PD-1 → cisplatin treatment group, but to a lesser extent than that in monotherapy treatment with anti-PD-1 (Supplementary Fig. S7B).

**Figure 6:**
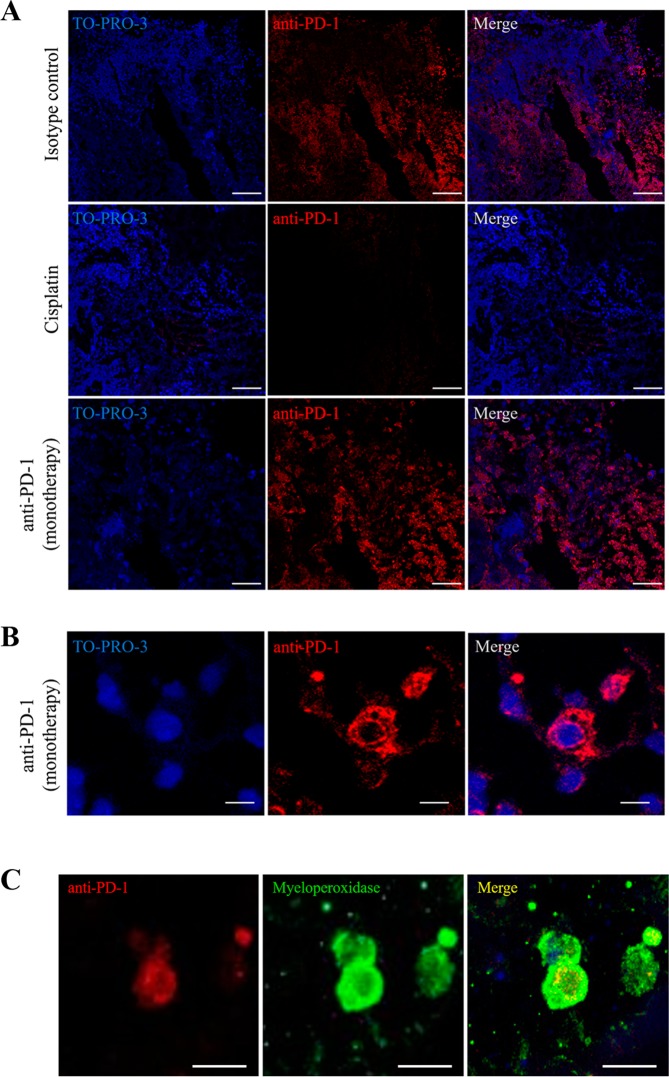
Detection of anti-PD-1 binding in tumors from the control, cisplatin and anti-PD-1 monotherapy groups in our patient-derived xenograft (PDX) model. (**A**) Representative confocal microscopy images of anti-PD-1 bound to PD-1 (red) in tumors from the control, cisplatin, and anti-PD-1 monotherapy groups. Nuclei were stained with TO-PRO-3 (blue). Right panels show merged images. Scale bars, 100 µm. **(B)** Representative images of the location of anti-PD-1 bound to the membrane and the cytoplasm of infiltrated cells and in necrotic areas of tumors in the anti-PD-1 monotherapy group. Nuclei were stained with TO-PRO-3 (blue). Right panel shows a merged image. Scale bars, 10 µm. **(C)** Neutrophils and anti-PD-1 binding sites. Detection of anti-PD-1 (red) and myeloperoxidase (green) in tumor infiltrate cells in a PDX tumor by confocal microscopy. Shown are representative images of expression and localization. Right panel shows merged images of co-localization (yellow). Scale bars, 20 µm.

Our results so far suggest that neutrophils are the effector cells in response to anti-PD-1 treatment in the PDX NOD-SCID gamma model. To address the underlying mechanisms implicated in this response and the target “location” of the therapy, we used IHC to analyze human PD-1 and PD-L1 expression, with the aim of identifying the anti-PD-1 target and its ligand in treated and untreated tumor samples. However, the commercial antibodies showed no reactivity in our assays (data not shown). We had more staining success with anti-PD-1 treatment antibody as a primary antibody, which was revealed with an anti-hIgG secondary antibody. We detected PD-1-like binding sites in tumor sections of the isotype control and the anti-PD-1 treatment groups, but not in the cisplatin group (Fig. 6A). The anti-PD-1 labeling was localized to the cell membrane surrounding the necrotic areas within the tumor and was more delocalized in the cytoplasm (Fig. 6B). The anti-PD-1 monotherapy tumor showed stronger labeling than the tumors from the other experimental groups (Figs. 6A,B). Double immunofluorescence staining using the anti-PD-1 treatment antibody together with the anti-MPO antibody identified neutrophils as the target cells for anti-PD-1 (Fig. 6C). Finally, we assessed anti-PD-1 treatment antibody binding to the original patient tumor tissue by immunofluorescence (Fig. S7C in Supplementary Material), which indicated the presence of immunotherapy binding sites, presumably PD-1 receptors. However, their expression in the tumor tissue of the patient could not be confirmed by conventional IHC using the available commercial anti-PD-1 antibody.

### Immunofluorescence experiments in tumors: Identification of anti-PD-1 binding sites in neutrophils

**Figure 7:**
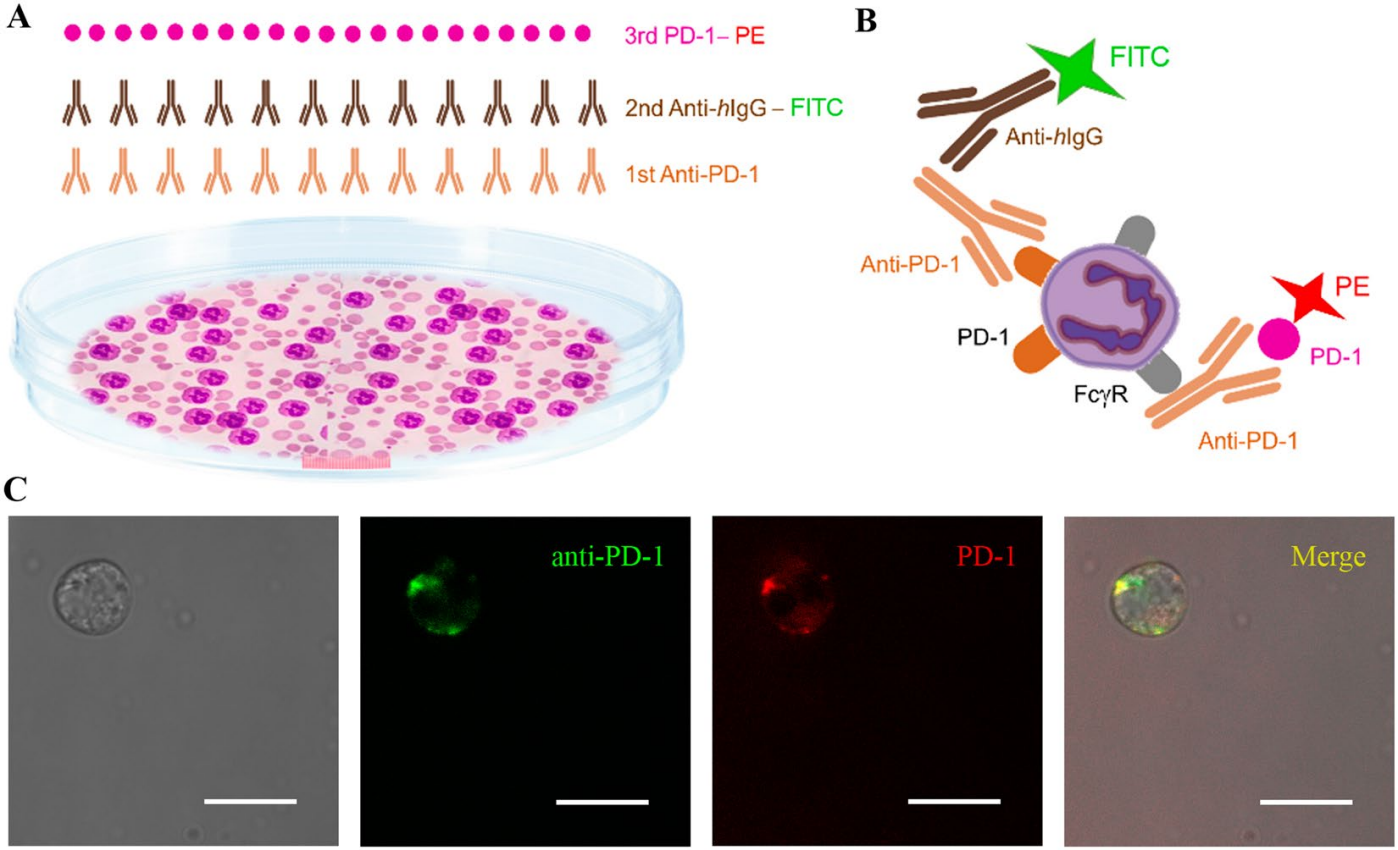
Anti-PD-1 antibody binding sites in the neutrophil membrane surface. (**A**) Schematic representation of the double immunofluorescence experiment. **(B)** Schematic representation of the results: the anti-PD-1 antibody binds to two different sites of the neutrophil membrane surface, PD-1 receptors and fragment crystallizable (Fc)-gamma receptors (FcγR). **(C)** Representative confocal microscopy images of anti-PD-1 bound to PD-1 receptor (in green) in insolated murine neutrophils. The PD-1 active protein (in red) is bound to anti-PD-1 which is in turn is attached to the neutrophil surface membrane through FcγR. Right panel shows merged images of co-localization (yellow) of both receptors in the neutrophil. Scale bars, 10 µm. Abbreviations: Fab, antigen-binding fragment; FITC, fluorescein isothiocyanate; PE, phycoerythrin.

The degree of the neutrophil purity achieved in whole peripheral blood from NOD-SCID gamma mice assessed by flow cytometry (CD11b+ Ly6G+ cells) was 99%. To identify the specific receptors through which anti-PD-1 binds to the neutrophil membrane surface, a double immunostaining was applied to analyze both FcγR and PD-1 receptors by confocal microscopy (schematic representation of the design shown in Fig. 7A). We observed that *(i)* anti-PD-1 (shown in green colour) bound to the PD-1 receptors located in the cell membrane surface of isolated murine neutrophils, through its Fab region; and *(ii)* PD-1 protein (red colour) bound to anti-PD-1 which in turn also bound to the cell surface membrane through FcγR (co-location of both signals shown in yellow) (see Figs. 7B [schematic representation of results], 7C [confocal images] and Fig. S8 in Supplementary Material). In the control experiment there was total absence of immunofluorescence staining. These data indicate that neutrophils might be potential targets of treatment with anti-PD-1 immunotherapy.

## Discussion

Immunotherapies such as PD-1 inhibitors in lung cancer have resulted in unprecedented improvements in patient survival^1,2^. However, these therapies do not benefit all patients and many issues remain to be worked out, including the mechanisms of action and the possible effector function of immune cells from non-lymphoid lineages. We based our study on an early-stage NSCLC PDX model, which allowed us to develop sensitivity assays to anti-PD-1 therapy in the absence of a reconstituted immune system in NOD-SCID gamma mice. An antitumor effect was observed in animals that received anti-PD-1 treatment, alone or in combination with cisplatin, possibly due to a mechanism independent of T lymphocytes, which has not previously been described. The anti-PD-1 treatment induced myeloid cell mobilization to the tumor, together with the production of exudates compatible with an acute inflammatory reaction mediated by murine PMNs, specifically neutrophils. Accordingly, we have provided preliminary evidence for a new immunotherapy mechanism, suggesting a potential cytotoxic action of neutrophils as PD-1 inhibitor effector cells that might be responsible for tumor regression by necrotic extension.

Only about 20% of patients with NSCLC receiving immunotherapy respond to treatment^8,9,21^. Because the purpose of this study was to investigate immunotherapy sensitivity in the absence of lymphocytes, an anti-PD-1 responder PDX line was selected. Of note, a non-responder line (PDX6) was also studied. As expected, chemotherapy (cisplatin) alone produced a good response, with tumor regression >50% in terms of necrotic extension, and stabilized the tumor graft growth rate along the treatment. By contrast, the response to anti-PD-1 therapy, alone or sequentially combined with cisplatin, was paradoxical, and led to an increase in tumor growth rate (in the anti-PD-1 phase) with large and friable tumors in some cases, which were associated with exudates containing inflammatory PMNs from areas of reactive necrosis. This phenomenon is reminiscent of unconventional responses of checkpoint inhibitor-based immunotherapy, such as pseudoprogression, which can be observed in patients´ tumors treated with this type of immunotherapy^22–24^. These ‘paradoxical’ or ‘unconventional’ responses are associated both with immune cell (PMNs and lymphocytes) recruitment and with the intratumoral inflammatory environment triggered by those cells^22,23^. We also found an inflammatory necrotic process accompanied by fluids and exudates in the concomitant chemo-immunotherapy (anti-PD-1 + cisplatin) treatment group, although the tumor growth rate and volume were reduced drastically. The best results in terms of tumor regression were found with the aforementioned concomitant treatment, with a necrotic index ~90% and a very reduced viable tumor component, which suggests a synergic effect of both therapies.

In view of the current clinical dilemmas of immunotherapy^25^, we used the PDX model to test whether there were differences in the application of sequential treatment with chemo-immunotherapy; that is, anti-PD-1 first and then cisplatin (anti-PD-1 → cisplatin) or *vice versa* (cisplatin → anti-PD-1). We also wanted to know if delivering treatment sequentially was more efficient than concurrently^25,26^. However, our results do not provide a basis for concluding that any of the options would have more benefits over the other. While anti-PD-1 → cisplatin combination did reduce tumor volume and increase necrosis as compared with the isotype control group, this effect is probably explained by the chemotherapy administration. Nonetheless, sequential treatments were inferior to the concomitant/concurrent administration of chemo-immunotherapy in terms of tumor regression.

Theoretically, infiltrating cytotoxic T lymphocytes are the anti-tumor effector cells in anti-PD-1 therapy^3,7^. NOD-SCID gamma mice, the model used in this study, are characterized by the absence of T and B lymphocytes and NK cell functionality. However, the innate immune system is functionally active in these animals. Our results show that the original human infiltrate was replaced by a murine tumor infiltrate, which is expected in this model^27,28^. Indeed, we found a rich murine myeloid component in the tumor stroma of mice treated with anti-PD-1. In this context, it is known that some myeloid cells such as dendritic cells^29^, macrophages^29,30^ and neutrophils^31,32^, have important roles as mediators in the tumor microenvironment. In addition, these cells could contribute to the anti-tumor action, blocking immune checkpoints^29,33^. The recruitment of myeloid cells during immunotherapy treatment has been related to an anti-tumor response^29,34^ with cytotoxic capacity as their main mechanism^29,35^. It is possible that these cells replace cytotoxic lymphocytes in response to anti-PD-1^3,7^ under immunosuppression conditions, increasing their tumor recruitment in tumors treated with anti-PD-1, as suggested here.

The mechanisms by which intra-tumor myeloid cells function are not clear, particularly the duality of mechanisms regarding the polarization towards pro/anti-inflammatory and pro/anti-tumor capacity in tumor-associated macrophages^30^. Indeed, we are beginning to realize that there are two different populations of tumor-associated neutrophils (TANs), some of them anti-tumor (N1) and others pro-tumor neutrophils (N2)^31,32,36^. This phenomenon is not well understood in detail, but it seems to involve a balance between pro- and anti-tumor populations^31,32,37^.

We detected inflammatory exudates in tumors from the anti-PD-1 groups (treated with anti-PD-1 alone or in combination with chemotherapy) associated with large necrotic areas containing destructed tissue and PMNs, mostly neutrophils. The clinical significance of this is controversial because some studies associate a tumor infiltrate rich in neutrophils with poor prognosis^31,33,38^, while others establish a good association between this infiltrate and a positive response to immunotherapies^33,39^. Our data suggest that neutrophils located in the necrotic areas of tumors from the anti-PD-1 groups are active, and respond to anti-PD-1 through NO production and the consequent formation of nitrotyrosine, a marker of oxidative damage^31,32,36,39^. The most probable mechanism would be the known cytotoxic action of TANs, especially of those with an N1 phenotype^31,32,36,39^.

The necrotic areas evident in the anti-PD-1 treatment histologies are very different morphologically from those produced by cisplatin, indicating that different mechanisms of cell death exist between treatments. Apoptosis is the main mechanism of action of cisplatin^40,41^, although we found no evidence of apoptosis in the tumors from the anti-PD-1 treatment groups (data not shown). Overall, the finding of strong nitrotyrosine labeling in necrotic areas of tumors from the anti-PD-1 treatment group, and its co-localization with MPO, an accepted marker of neutrophils, suggests that infiltrated neutrophils are responsible for the mechanism of cell death by cytotoxicity and NO production, phenomena usually associated with acute inflammation foci^31,42^. This finding was also observed in the sequential treatment groups, but was less marked.

The results of the present study suggesting a role of neutrophils in response to immunotherapy are novel. The action principle of targeted therapies consisting of monoclonal antibodies is their specific binding to a particular antigen (protein) and its blockade^2,43,44^. In the isotype control group, anti-PD-1 binding sites were detected indirectly in the membrane of some cells as well as in delocalized locations (in the cytoplasm of other cells). Because we used nivolumab for this analysis, we presume that PD-1 was blocked in murine cells. This seems in accordance with aforementioned paradoxical or unconventional responses to immunotherapy and contradicts the IHC results in the PDX tumors with commercial antibodies, which were negative for this staining. By contrast, in the immunofluorescence experiments where anti-PD-1 was used as a primary antibody, we found PD-1 receptors in tumor stroma. According to our results in tumors whose treatments included cisplatin, the anti-PD-1 did not bind to any specific site in the tumor, suggesting that cisplatin may be destroying cells that expressed the PD-1 receptor or prevents the expression of PD-1 receptor. The detection of anti-PD-1 in tumors that received anti-PD-1 treatment was increased, indicating that this therapy stimulates the expression of its own target^45,46^.

On the other hand, neutrophils, both infiltrated or located in necrotic areas of tumors treated with anti-PD-1, appear to express the PD-1 receptor on their membrane. Indeed, our immunofluorescence experiments indicated binding of anti-PD-1 to PD-1 receptors in these cells. To our knowledge, there is no precedent for direct neutrophil activation by an anti-PD-1 monoclonal antibody. Preliminary data has suggested that PD-L1 and, occasionally, PD-1 can be expressed on the surface of neutrophils, but there must be T lymphocyte mediation^47,48^, which is not the case in our model. Thus, although more research is needed, especially in patients, our results are novel and suggest that neutrophils might be potential targets of treatment with anti-PD-1 immunotherapy.

**Figure 8:**
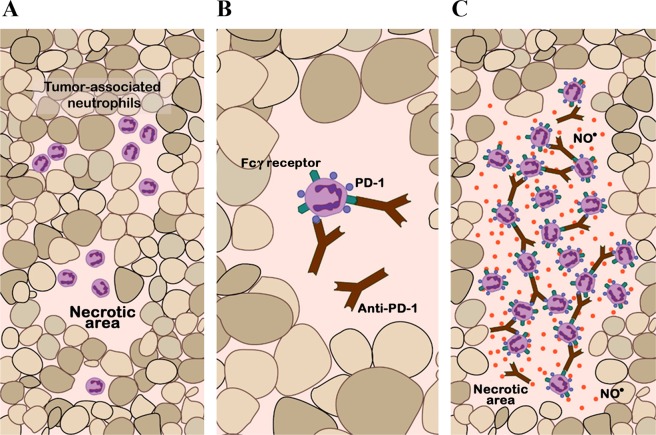
Schematic representation of the possible mechanism of action of neutrophils. (**A**) Basal situation of a patient-derived xenograft. Necrotic areas are represented in a squamous lung cancer, in which an immune infiltrate of murine myeloid cells, including neutrophils, is evident. **(B)** Intra-tumor distribution of anti-PD-1 therapy. The anti-PD-1 antibody can bind to the PD-1 receptor on the neutrophil membrane; in addition, anti-PD-1 can recognize fragment crystallizable (Fc) gamma receptors (FcγR) and binds to their antibody Fc region. **(C)** Acute inflammatory reaction and neutrophil-mediated opsonization. Following on from (B), there is an inflammatory chain reaction and neutrophils acquire cytotoxic capacity and release nitric oxide (red circles in the figure), resulting in more extensive necrotic areas and subsequent tumor regression.

Based on our results and while keeping in mind the limitation that the study was done in a murine PDX model and in fact in only one immunotherapy-responder line of PDX mice with a small number of mice in each experimental group, neutrophil action appears to be the consequence of several processes (shown in Fig. 8), that would occur acutely as a ripple effect with the production of NO, causing necrotic expansion. One process would involve the direct activation of neutrophils by the binding of anti-PD-1 to PD-1 receptors located on the neutrophil surface and another process would be the binding of the anti-PD-1 antibody to FcγR, as previously suggested elsewhere^33,49^ and corroborated here. It has been described that neutrophils can perform powerful and fast cytotoxic functions (known as antibody-dependent cellular cytotoxicity) in the presence of monoclonal antibodies against tumor cells^36,50^. The neutrophil antitumor effector function mediated by Fc is not a well understood mechanism, but it is known that it could include opsonization and death mediated by necrosis^33,49,51,52^, as shown here. Moreover, neutrophils, the most abundant human leukocytes^53^, are the first to arrive during an inflammatory and infectious episode^53^, performing powerful cytotoxic functions^31,36,39^. Neutrophil response, as a component of innate immunity, is faster than the adaptive immune response. Therefore, it might be possible that neutrophils have a relevant role in antitumor therapies to reinforce immunosurveillance, such as immune checkpoint blockade. In this regard, it must be kept in mind that a major limitation of our study is that we did not determine the relative impact of neutrophils − *vs* the main therapy effectors, T lymphocytes − to the overall anti-PD-1-induced antitumoral response in a ‘real’ (*i.e*., immunocompetent) setting.

PDX models are pertinent to predict patients’ response to different therapies^54,55^ and they are the most utilized models to evaluate drugs in late pre-clinical trials or to select personalized therapy^54,56^. Indeed, the disease of the patient was reproduced in the PDX line analyzed in this study, maintaining the histopathological features of the original tumor^55,56^. Our mouse model is based on immunodeficient mice lacking an adaptive immune system, which is suitable to study lymphocyte-independent mechanisms. Indeed, a critical aspect in the selection of the PDX model was the absence of a competent immune system; however, this might limit the generalizability of the results because the tumor microenvironment is not reliably reproduced^56,57^. In addition, although there is some debate, the tumor stroma from the donor patient is replaced by murine stroma, and completely disappears after several passages^56,58^. It should also be considered that although our results suggest that myeloid cells (neutrophils, specifically) are effectors of anti-PD-1, there are some differences between murine and human neutrophils^59^.

In summary, our findings suggest a novel mechanism of immunotherapy involving the potential cytotoxic action of neutrophils as PD-1 inhibitor effector cells responsible for tumor regression by necrotic extension. Further research is needed to corroborate the important role of these cells in immunosurveillance against tumors, such as immune checkpoint blockade, particularly using immunocompetent models.

## Supplementary information

Supplementary Information.

Supplementary Figure Legends.
