# Supplementary material for: Effects of anti-PD-1 immunotherapy on tumor regression: insights from a patient-derived xenograft model
Source: Sci Rep. 2020 Apr 27;10:7078. doi: 10.1038/s41598-020-63796-w (PMC7184589; doi:10.1038/s41598-020-63796-w)
Supplement: Supplementary file 1 — Supplementary Information. [file 41598_2020_63796_MOESM1_ESM.pdf]

SUPPLEMENTARY MATERIAL

Research Article

TITLE

Effects of anti-PD-1 immunotherapy on tumor regression: insights from a patient-derived xenograft model.

AUTHORS

Asunción Martín-Ruiz[1,2], Carmen Fiuza-Luces[3], Esther Martínez-Martínez[1,4], Clemente F. Arias[5], Lourdes Gutiérrez[1], Manuel Ramírez[6], Paloma Martín-Acosta[7], Maria José Coronado[8], Alejandro Lucia[2,3] and Mariano Provencio[1*].

***Correspondence:** Dr. Mariano Provencio, mprovenciop@gmail.com

SUPPLEMENTARY MATERIAL

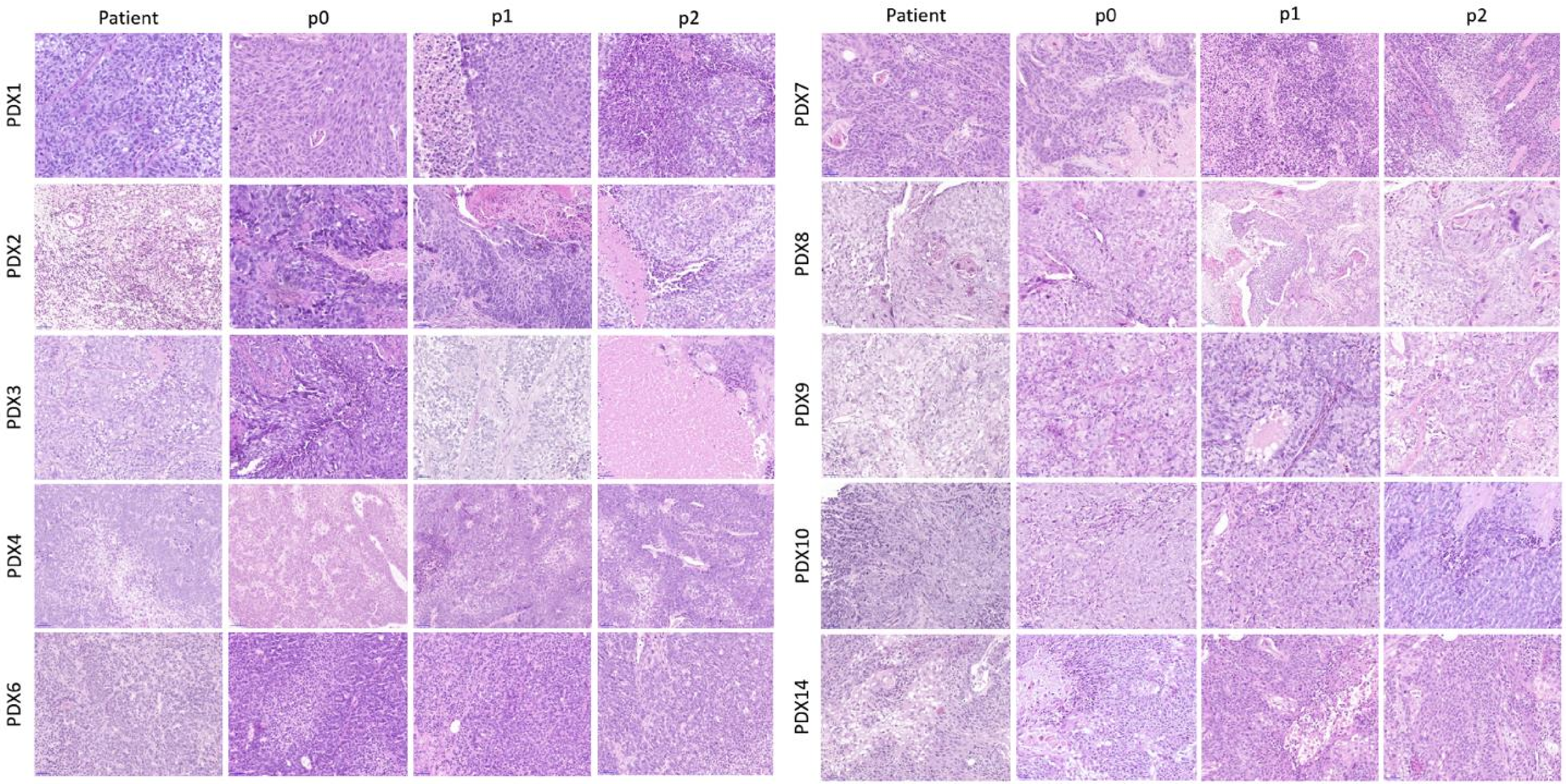
Patient
p0
p1
p2
Patient
p0
p1
p2
PDX1
PDX2
PDX3
PDX4
PDX6
PDX7
PDX8
PDX9
PDX10
PDX14

Figure S1

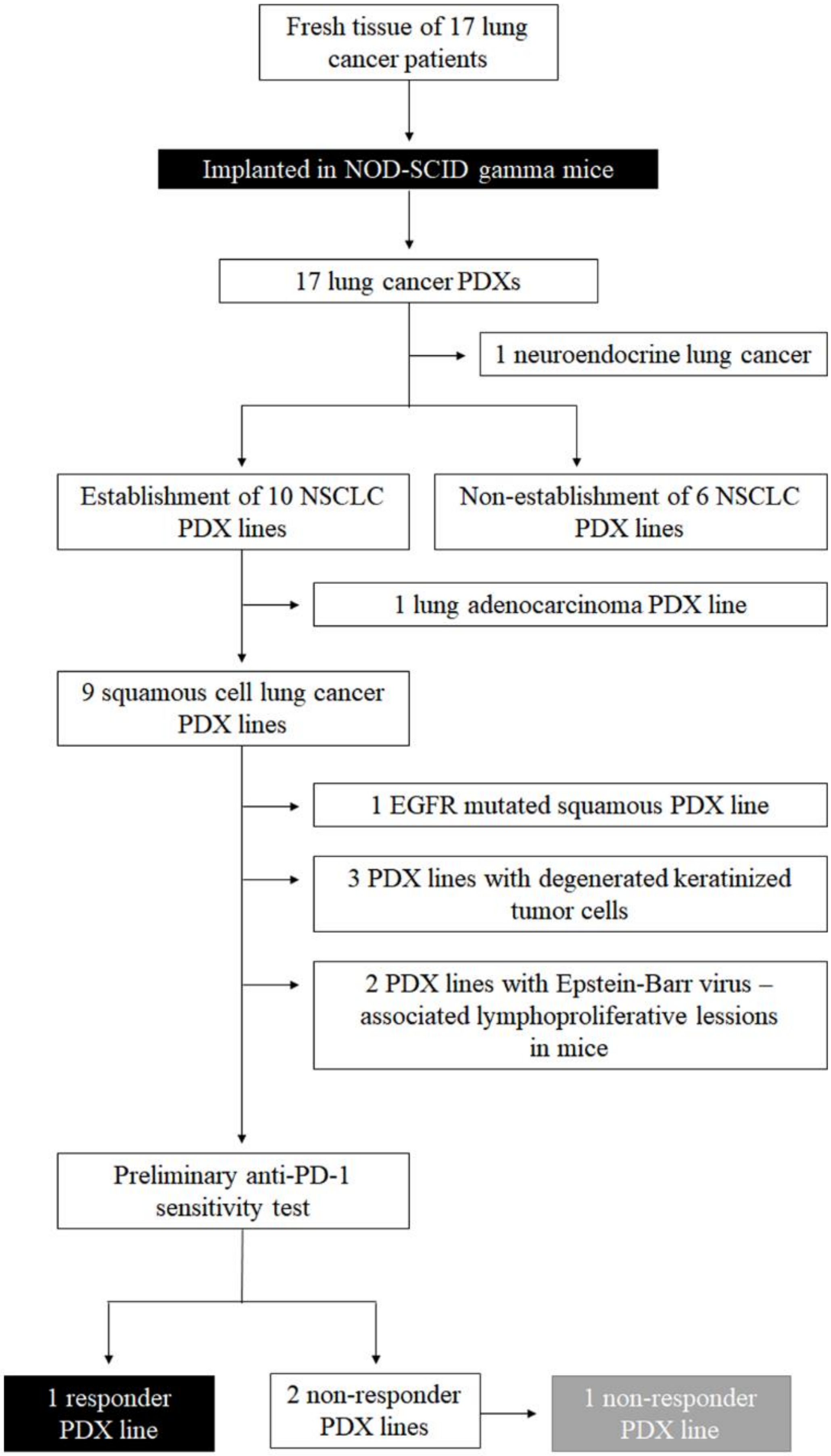

Fresh tissue of 17 lung cancer patients
Implanted in NOD-SCID gamma mice
17 lung cancer PDXs
1 neuroendocrine lung cancer
Establishment of 10 NSCLC PDX lines
Non-establishment of 6 NSCLC PDX lines
1 lung adenocarcinoma PDX line
9 squamous cell lung cancer PDX lines
1 EGFR mutated squamous PDX line
3 PDX lines with degenerated keratinized tumor cells
2 PDX lines with Epstein-Barr virus –associated lymphoproliferative lessions in mice
Preliminary anti-PD-1 sensitivity test
1 responder PDX line
2 non-responder PDX lines
1 non-responder PDX line

Figure S2

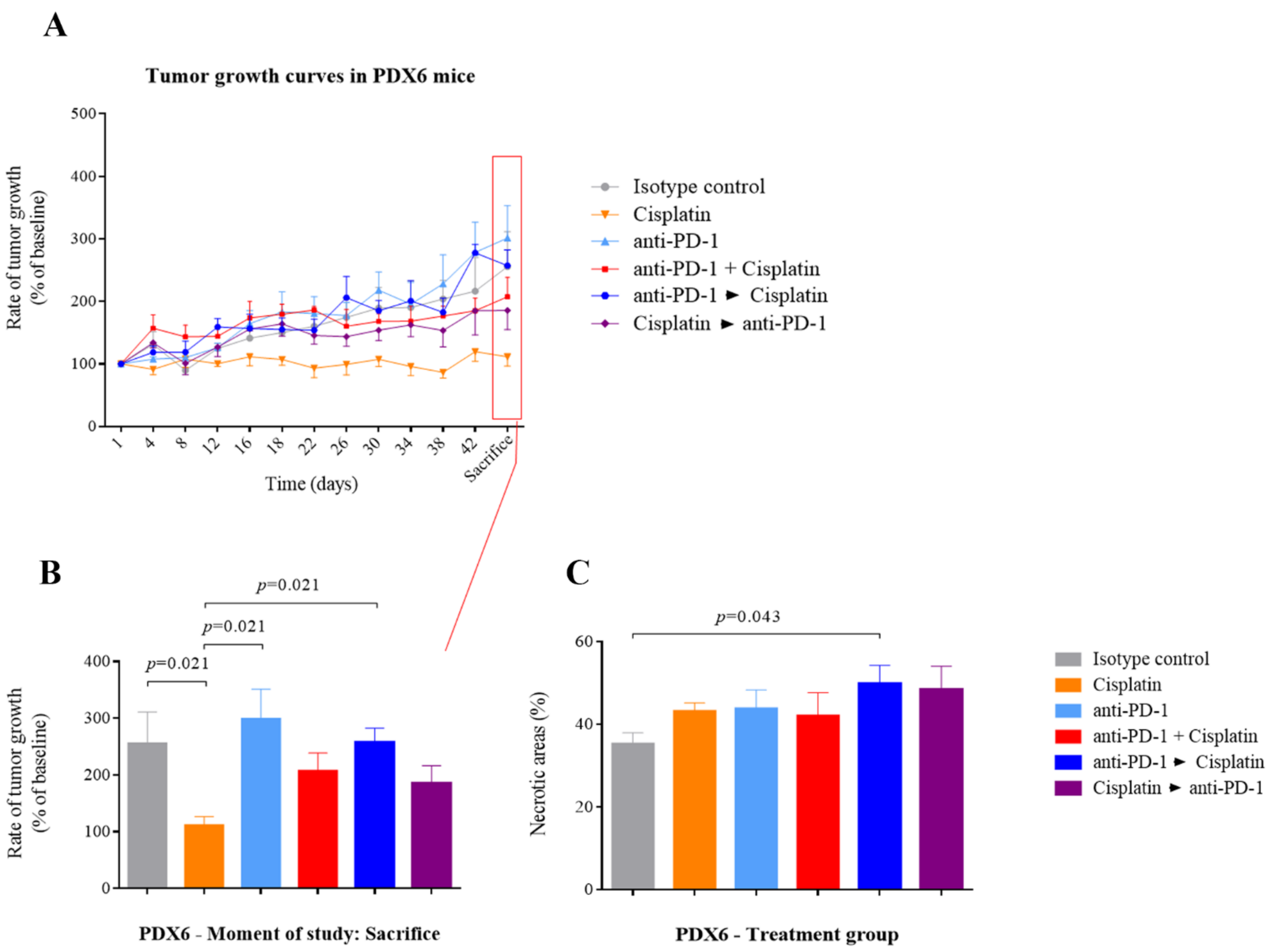
A
Tumor growth curves in PDX6 mice
Rate of tumor growth (% of baseline)
Time (days)
Sacrifice
Isotype control
Cisplatin
anti-PD-1
anti-PD-1 + Cisplatin
anti-PD-1 ► Cisplatin
Cisplatin ► anti-PD-1
B
p=0.021
p=0.021
p=0.021
Rate of tumor growth (% of baseline)
PDX6 - Moment of study: Sacrifice
C
p=0.043
Necrotic areas (%)
PDX6 - Treatment group

Figure S3

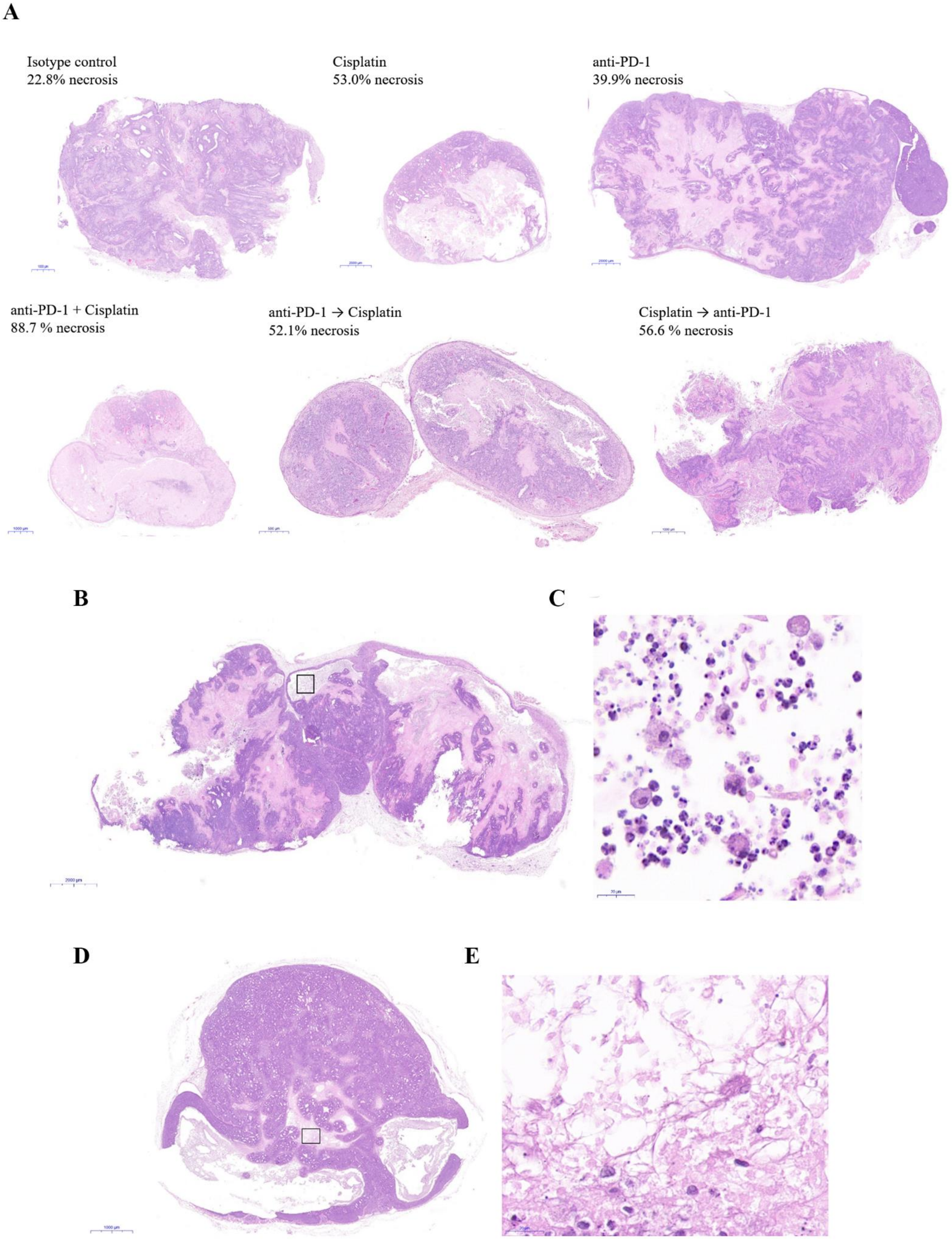
A
Isotype control
22.8% necrosis
Cisplatin
53.0% necrosis
anti-PD-1
39.9% necrosis
anti-PD-1 + Cisplatin
88.7 % necrosis
anti-PD-1 → Cisplatin
52.1% necrosis
Cisplatin → anti-PD-1
56.6 % necrosis
B
C
D
E

Figure S4

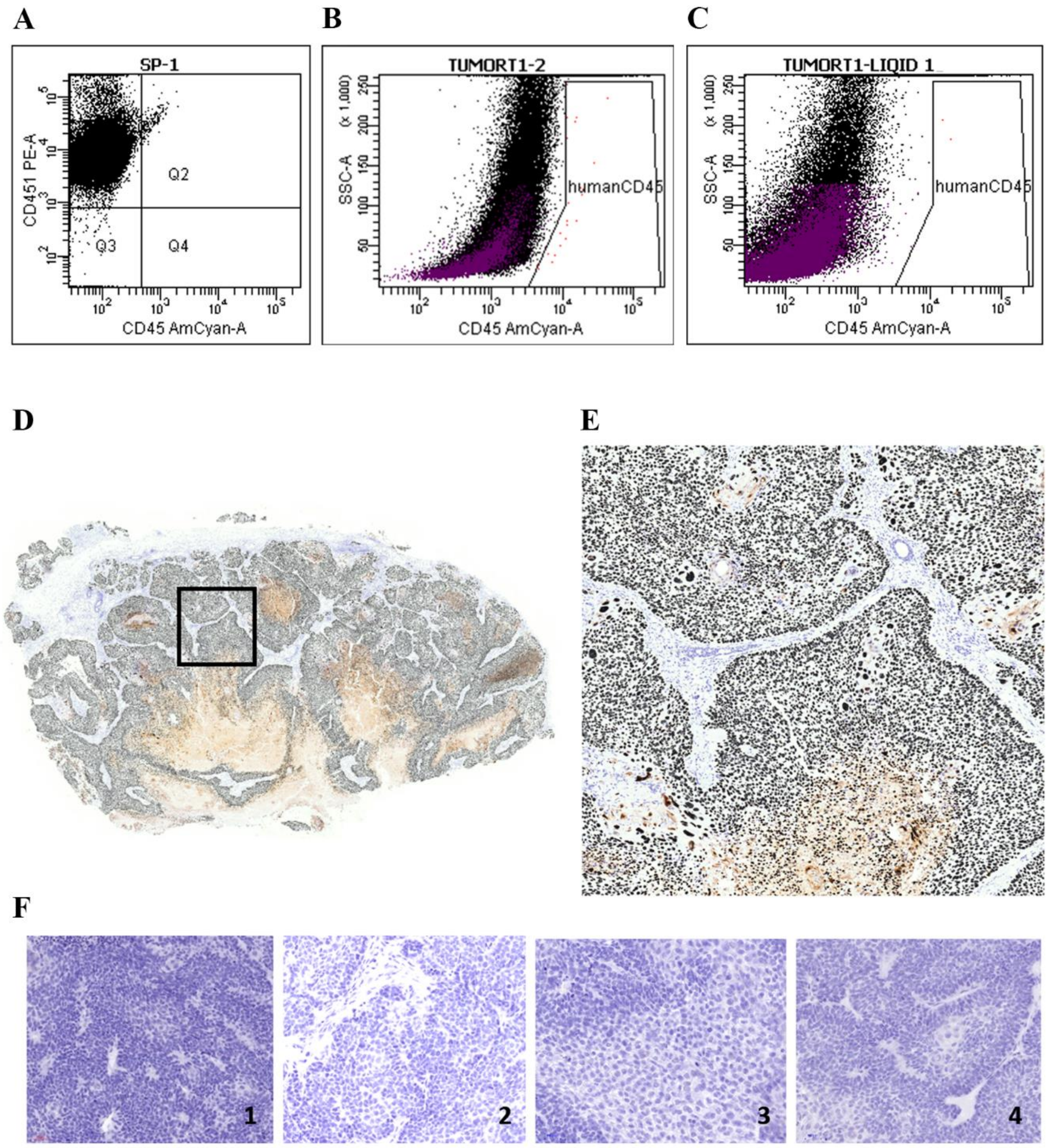
A
B
C
SP-1
TUMORT1-2
TUMORT1-LIQID 1
CD451 PE-A
CD45 AmCyan-A
SSC-A
(x 1,000)
Q2
Q3
Q4
humanCD45
D
E
F
1
2
3
4

Figure S5

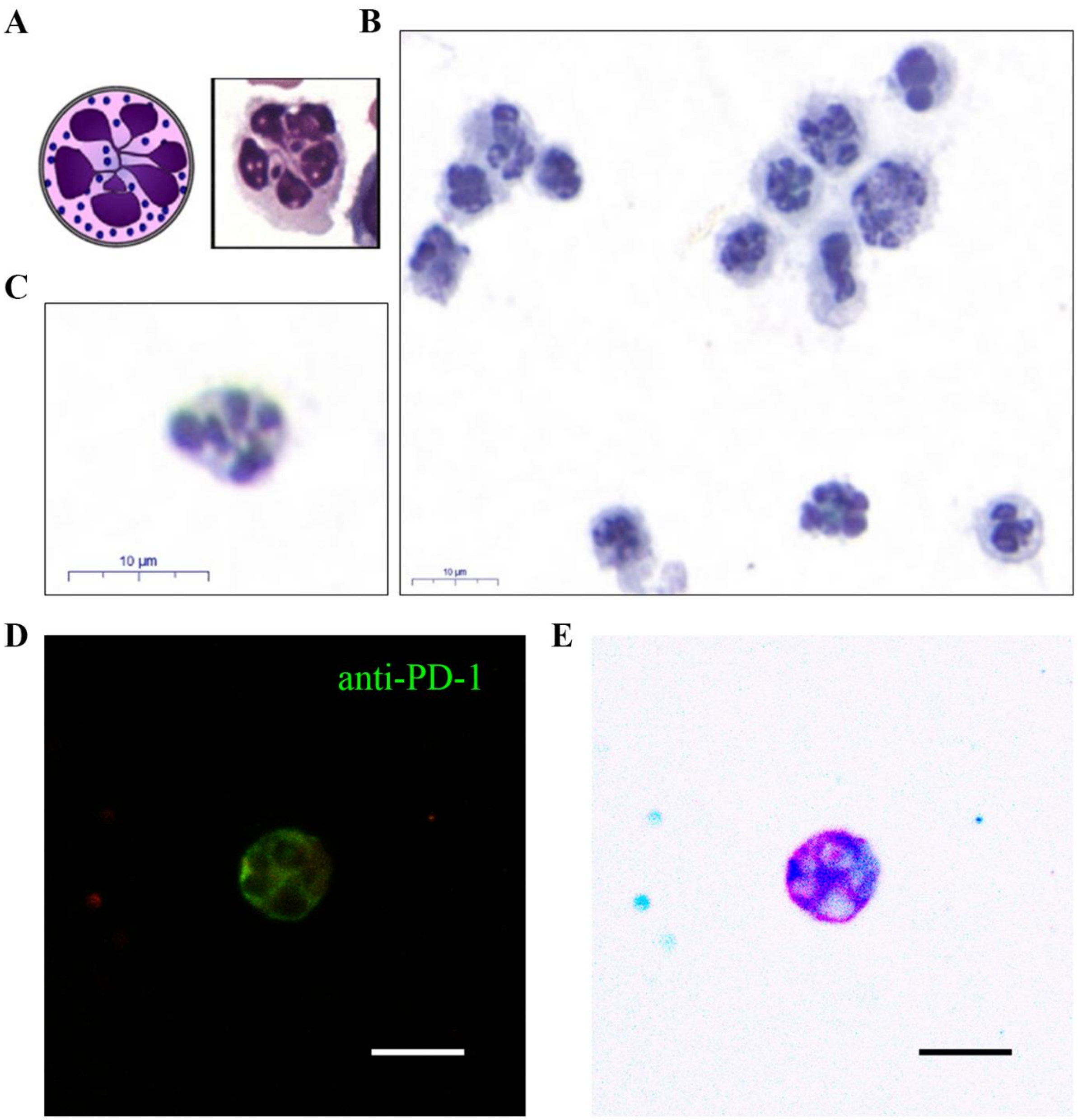

Figure S6

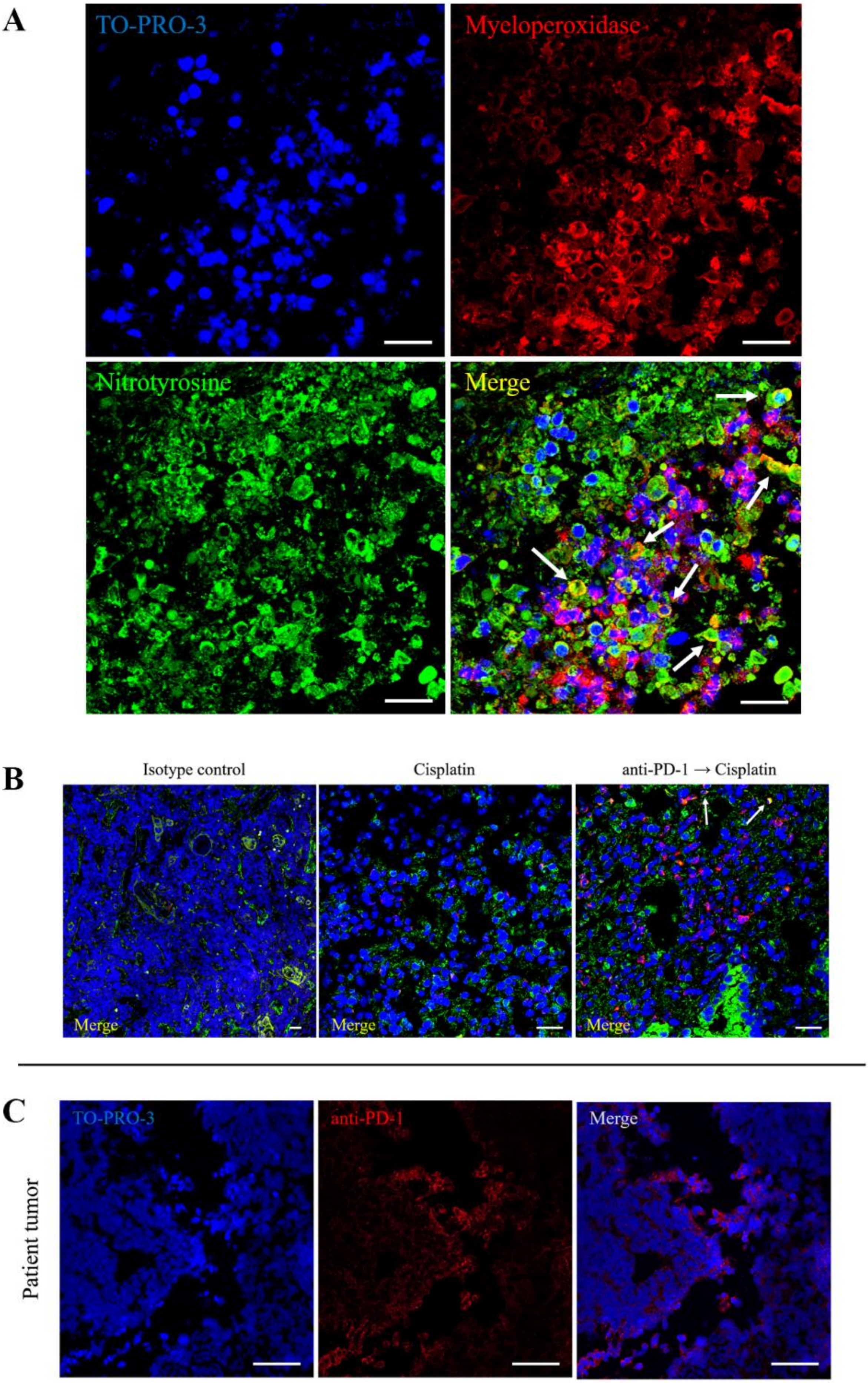
A
TO-PRO-3
Myeloperoxidase
Nitrotyrosine
Merge
B
Isotype control
Cisplatin
anti-PD-1 → Cisplatin
Merge
Merge
Merge
C
TO-PRO-3
anti-PD-1
Merge
Patient tumor

Figure S7

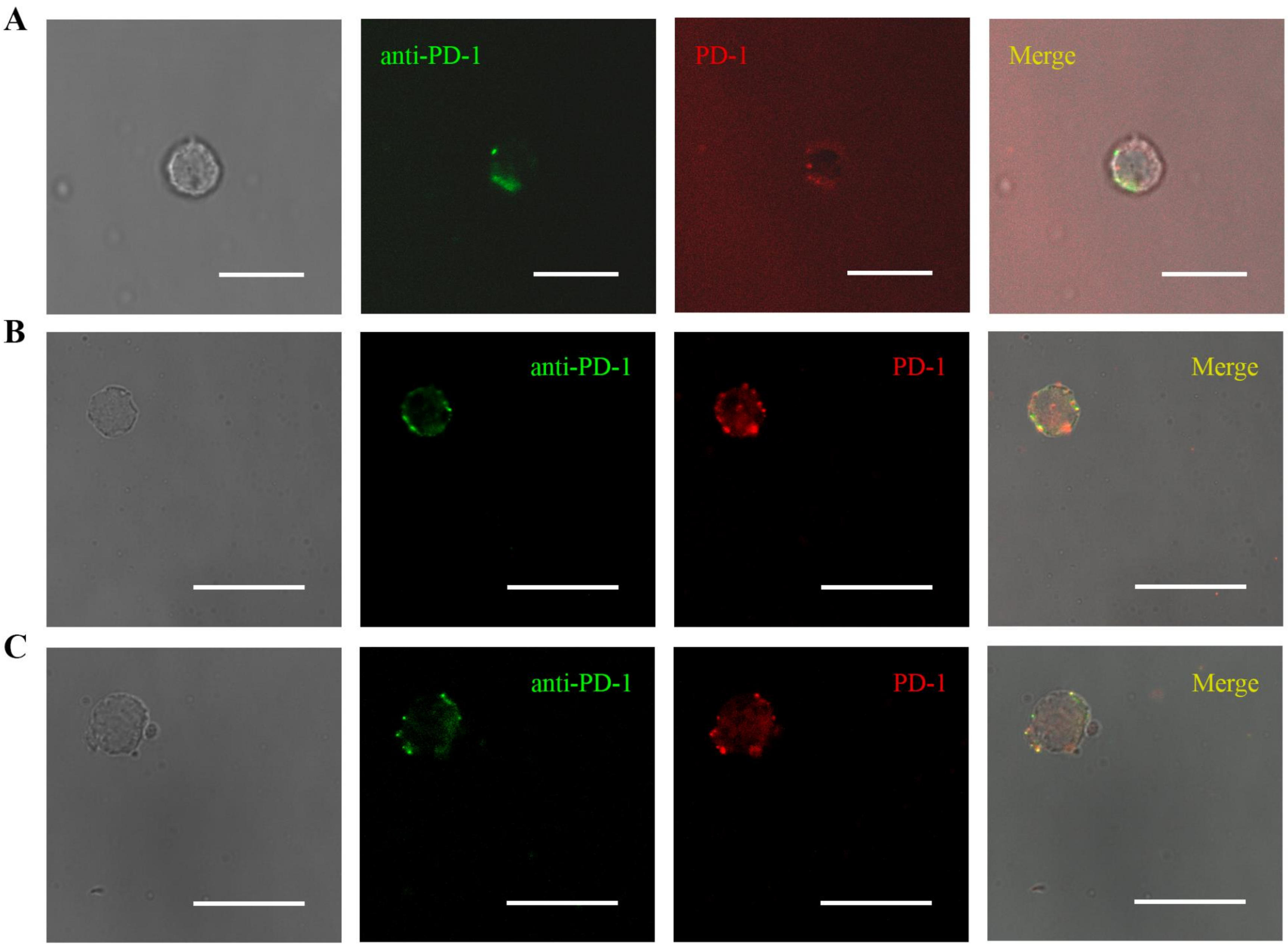

Figure S8

Supplementary text file S1

Establishment of a human squamous NSCLC PDX model

Female 8-week-old NOD-SCID gamma (NOD.Cg-*Prkdc*^{scid} *Il2rg*^{tm1Wjl}/SzJ) mice were purchased from Charles River Laboratories (Chatillon-sur-Chalaronne, France). Mice were transplanted with a direct surgically obtained tumor sample from patients with NSCLC using the following protocol. The specimen was sliced into fragments (~3 mm × 2 mm) and two pieces were implanted subcutaneously into the flanks (bilateral grafts) of each host mouse (passage 0 or p0) under inhalation anesthesia (sevoflurane in oxygen, Sevorane). After transplant, tumor growth was monitored and, whenever palpable, the volume was measured with a caliper (AA846R, Aesculap AG, Tuttlingen, Germany) twice weekly using the following formula: $(4\pi/3) \times (w/2)^2 \times (l/2)$, where w = width and l = length. When the tumor volume reached ~1 cm^3, the mice were sacrificed by CO_2 inhalation. A portion of the harvested tumor was used for phenotype and molecular analyses to verify that the xenograft model was histopathologically stable, and another portion was harvested and serially re-engrafted to maintain the *in vivo* PDX line during subsequent passages (termed p1, p2, etc.). Several NSCLC PDX lines were established, and those lines fulfilling the following criteria were selected: squamous histology, not having driver mutations, and maintenance of the original tumor characteristics throughout the passages.

Preliminary test of response to anti-PD-1 therapy

To evaluate the response of the PDX model to immunotherapy, a preliminary test was performed based on changes to tumor volume, which would allow for the detection of responders *versus* non-responders. Groups of three mice (p1) were administered with anti-PD-1 therapy (nivolumab [Opdivo®, Bristol-Myers Squibb, Princeton, NJ, USA is a fully human immunoglobulin G4 (hIgG4, Crown Bioscience, Inc. [Santa Clara, CA, USA]) PD-1 immune checkpoint inhibitor antibody that disrupts the interaction of PD-1 with its ligands PD-L1 and PD-L2, blocking the immune response], 150 μg) or an equivalent dose of an irrelevant hIgG4 by intra-peritoneal (i.p.) injection twice weekly for 3 consecutive weeks. Responder mice were selected and maintained through consecutive passages.

Supplementary Table S1. Primary antibodies used for different immunodetection techniques

Antibody	Supplier	Clone	Type	Species	Uses	System	Dilution
TTF-1	Dako	8G7G3/1	mAb, mouse	Human	IHC	Omnis Dako	1:200
CD56	Dako	123C3	mAb, mouse	Human	IHC	Omnis Dako	1:50
Synaptophysin	Dako	DAK-SYNAP	mAb, mouse	Human	IHC	Omnis Dako	*Ready-to-use*
Napsin-A	Novocastra	IP64	mAb, mouse	Human	IHC	Leica	1:500
p40	Dako	BC28	mAb, mouse	Human	IHC	Ventana (Roche)	1:500
p63	Dako	DAK-p63	mAb, mouse	Human	IHC	Omnis Dako	*Ready-to-use*
CK-5/6	Dako	D5/16 B4	mAb, mouse	Human	IHC	Omnis Dako	*Ready-to-use*
CD45	Dako	2B11 + PD7/26	mAb, mouse	Human	IHC	Omnis Dako	*Ready-to-use*
CD3	Dako	GA503	pAb, rabbit	Human	IHC	Omnis Dako	*Ready-to-use*
CD4	Dako	4B12	mAb, mouse	Human	IHC	Omnis Dako	*Ready-to-use*
CD20cy	Dako	L26	mAb, mouse	Human	IHC	Omnis Dako	*Ready-to-use*
PD-1	Cell Marque	NAT105	mAb, mouse	Human	IHC	Ventana (Roche)	1:50
PD-L1	BioLegend	29E.2A3	mAb, mouse	Human	IHC	Manual	1:200
Nitrotyrosine	Abcam	HM.11	mAb, mouse	Human/mouse	IF	Manual	1:25
Myeloperoxidase (MPO)	R&D Systems	AF3174	pAb, rabbit	Human/mouse	IF	Manual	1:25
Nivolumab (anti-PD-1)	Bristol-Myers Squibb	-	humanized mAb	Human/mouse?	IF	Manual	1:200

Immunohistochemistry (IHC), immunofluorescence (IF), monoclonal antibody (mAb), polyclonal antibody (pAb). Markers of NSCLC immunophenotype panel shaded in light gray; other immunohistochemistry markers are shaded in dark gray and immunofluorescence markers are non-shaded

Supplementary Table S2. List of antibodies used for flow cytometry analysis

Protein	Clone	Brand
hCD33	HIM3-4	Becton Dickinson–PharMingen
hCD4	SK3	Becton Dickinson–PharMingen
hCD56	NCAM16,2	Becton Dickinson–PharMingen
hCD19	HIB19	Becton Dickinson–PharMingen
hCD8	SK1	BioLegend
hCD3	UCHT1	Becton Dickinson–Horizon
hCD45	2D1	Becton Dickinson–PharMingen
7-AAD	–	BioLegend
mCD45.1	A20	BioLegend
mLy6G	1A8	BioLegend
mNK1.1	PK136	BioLegend
mCD11B	M1/70	BioLegend
mCD3	145-2C11	BioLegend
mCD45	30-F11	BioLegend

Supplementary Table S3. Antibodies per tube and per tumor homogenate sample used for flow cytometry analysis based on fluorophore labeling.

	FITC	PE	PerCP	PE-Cy7	APC	APC-Cy7	PB	AC
**Peripheral blood tube**	hCD33	mCD45.1	hCD4	hCD56	hCD19	hCD8	hCD3	hCD45
**Tumor tube 1**	hCD4	mCD45.1	7-AAD	hCD56	hCD19	hCD8	hCD3	hCD45
**Tumor tube 2**		mLy6G	7-AAD	mNK1.1	mCD11b	mCD3		mCD45
**Isolated neutrophil tube**	mCD206	mCD11c	7-AAD	mF4/80	mCD11b	mLy6G	mLy6C	mCD45

Abbreviations: FITC (Fluorescein isothiocyanate); PE (Phycoerythrin); PerCP-CY7 (Peridinin chlorophyll protein); PE-Cy7 (Phycoerythrin cyanin 7); APC (Allophycocyanin); APC-Cy7 (Allophycocyanin-cyanin 7); PB (Pacific blue); AC (AmCyan).

Supplementary Table S4. Antibody conditions for immunofluorescence staining.

	Primary antibody			Secondary antibody		
		**Dilution**	**Supplier**		**Dilution**	**Supplier**
**Myeloperoxidase**	anti-MPO	1:25	R&D Systems	anti-goat IgG Alexa 546	1:500	Invitrogen
**Nitrotyrosine**	anti-Nitrotyr	1:25	Abcam	anti-mouse IgG Alexa 488	1:500	Invitrogen
**PD-1**	anti-PD-1	1:200	Bristol-Myers Squibb	anti-human IgG Alexa 546	1:500	Invitrogen
**Double immunofluorescence: Myeloperoxidase/Nitrotyrosine**	anti-MPO	1:25	R&D Systems	anti-goat IgG Alexa 546	1:500	Invitrogen
	anti-Nitrotyr	1:25	Abcam	anti-mouse IgG Alexa 488	1:500	Invitrogen
**Double immunofluorescence: Myeloperoxidase/PD-1**	anti-MPO	1:25	R&D Systems	anti-goat IgG Alexa 488	1:500	Invitrogen
	anti-PD-1	1:200	Bristol-Myers Squibb	anti-human IgG Alexa 546	1:500	Invitrogen
**Double immunofluorescence of isolated neutrophils: PD-1/FcγR**	anti-PD-1	1:20	Bristol-Myers Squibb	anti-human IgG–FITC	1:200	A. Menarini Diagnostics
	PD-1–PE	1/500	Abcam			
**Control double immunofluorescence of isolated neutrophils: PD-1/FcγR**	hIgG4	1:20	Crown Bioscience, Inc.	anti-human IgG–FITC	1:200	A. Menarini Diagnostics
	PD-1–PE	1/500	Abcam			

Supplementary Table S5. Clinical and pathologic characteristic of patients and passagable lung cancer

ID/No.	Gender	Smoking status	Age	Pathology	Localization	Grade / differenciation	Type	Mutation	Tumor-stage	Established Xenograft
PDX1	M	YES	78	SQC	RUL	Moderately	Infiltrating	NO	pT2a N0 L0 V0 R0 (IB)	YES
PDX2	M	YES	66	SQC	RUL	Poorly	Infiltrating / keratinized	NO	pT2a N0 L1 V1 R0 (IB)	YES
PDX3	M	YES	73	SQC	RUL	Poorly	Non-infiltrating	NO	pT2b N0 (IB)	YES
PDX4	M	YES	79	SQC	ML, RLL	Poorly	Infiltrating / basaloid	NO	pT2a N1 L1 V0 R0 (IIA)	YES
PDX5	M	YES	68	SQC	LLL	Moderately	Infiltrating	NO	pT4 N1 M0 (IIIA)	NO
PDX6	M	NO	62	SQC	LUL	Poorly	Infiltrating / basaloid	NO	pT1b N0 L0 V0 R0 (IA)	YES
PDX7	M	YES	71	SQC	RUL	Moderately	Infiltrating / keratinized	NO	pT3 N1 R0 (IIIA)	YES
PDX8	M	YES	74	SQC	RUL	Poorly	Infiltrating / keratinized	NO	pT2b N1 P0 V0 R0 (IIB)	YES
PDX9	M	YES	55	ADC	ML	Poorly	Infiltrating	NO	pT2a N0 P3 V2 R0 (IB)	YES
PDX10	M	YES	64	SQC	LUL	ns	Basaloid	NO	pT1b N0 M0 (IA)	YES
PDX11	F	YES	64	ADC	RUL	ns	Infiltrating	NO	pT2 N0 L1 V1 R0 (IB)	NO
PDX12	F	NO	80	ADC	RLL	ns	Infiltrating	Exon19Del	pT2a N0 M0 (IB)	NO
PDX13	M	YES	65	ADC	RUL	ns	Infiltrating	ALK/EML4	pT2 N2 M0 (IIIA)	NO
PDX14	M	YES	63	SQC	LUL	Poorly	Infiltrating / keratinized	G719X	pT3 N1 L1 V1 R0 (IIIA)	YES
PDX15	M	YES	36	ADC	LUL	ns	Infiltrating	NO	pT2a N0 (IB)	NO
PDX16	F	NO	60	NEC	LLL	Poorly	ns	NO	pT2a N0 L1 V0 R0 (IB)	YES
PDX17	M	NO	74	ADC	RLL	ns	Infiltrating	NO	pT2a N0 M0 L1 (IB)	NO

Abbreviations: M, male; F, female; SQC, squamous carcinoma; ADC, adenocarcinoma; NEC, neuroendocrine carcinoma; RUL, right upper lobe; LUL, left upper lobe; ML, middle lobe; RLL, right lower lobe; LLL, left lower lobe; ns, not specified.
