# Supplementary material for: Effects of anti-PD-1 immunotherapy on tumor regression: insights from a patient-derived xenograft model
Source: Sci Rep. 2020 Apr 27;10:7078. doi: 10.1038/s41598-020-63796-w (PMC7184589; doi:10.1038/s41598-020-63796-w)
Supplement: Supplementary file 2 — Supplementary Figure Legends. [file 41598_2020_63796_MOESM2_ESM.docx]

**SUPPLEMENTARY FIGURE LEGENDS**

**Supplementary Figure S1.** Phenotypic characterization panel of the PDX model. Hematoxylin and eosin staining of patients´ tumors and PDX tumor mice at passage 0 (p0), passage 1 (p2) and passage 2 (p2), (magnification ×200).

**Supplementary Figure S2.** Study flow diagram. Abbreviations: NSCLC, non–small cell lung carcinoma; PDX, patient-derived xenograft.

**Supplementary Figure S3**. Effect of different treatments on tumor size and on tumor necrosis in patient-derived xenograft PDX6 mice. (**A**) Tumor growth curves are expressed as a percentage of the change of initial tumor volume, which was considered as 100%. Each point represents a measurement day given as the mean ± SEM of the tumor volume of the mice of each group. (**B**) Mice were sacrificed after six weeks and the tumor volume was measured. Results are shown as mean ± SEM of the tumor volume of the mice of each group. Statistical significance is indicated (p≤0.05) and was analyzed by the Kruskal-Wallis non-parametric test. Post hoc between-group comparisons were made with the Mann-Whitney test. (**C**) The necrosis index is expressed as a percentage of necrotic areas. Values represent the percentage (mean ± SEM). Statistical significance is indicated (p≤0.05) and was analyzed by the Kruskal-Wallis non-parametric test. Post hoc between-group post hoc comparisons were made with the Mann-Whitney test.

**Supplementary Figure S4**. Tumor PDX4 histologies and inflammatory response associated with tumor regression in response to anti-PD-1 treatments in PDX4 and PDX6. (**A**) Selection of representative histological hematoxylin and eosin-stained images (×1) of each PDX4 treatments group, showing evident necrotic areas. (**B**) Representative hematoxylin and eosin-stained image of a tumor from a PDX4 mouse treated with anti-PD-1 → cisplatin (sequential). Necrotic areas showing polymorphonuclear cells, macrophages and dead epithelial cells, ×1. (**C**) Zoomed image of the necrotic zone marked with a square in panel B, where polymorphonuclear cells are observed, ×100. (**D**) Representative hematoxylin and eosin-stained image of a tumor from a PDX6 mouse treated with anti-PD-1. Necrotic areas not showing polymorphonuclear cell infiltration, only remnants of dead epithelial cells, ×1. (**E**) Zoomed image of the necrotic zone marked with a square in panel D, ×100.

**Supplementary Figure S5.** Human cells in xenografts. (**A**) Flow cytometry analysis of the immune component in blood and in tumor homogenates. Representative dot plot showing human CD45 (hCD45) *versus* murine CD45 (mCD45.1) staining of peripheral blood of a PDX4 mouse. (**B**) Representative flow cytometry analysis of homogenized tumors, using hCD45. Cells were stained with antibodies against human, hCD45 (red) and murine, mCD45 (purple) leukocytes. (**C**) Representative flow cytometry analysis of the inflammatory component from fluid collected from a PDX4 tumor treated with anti-PD-1, as in B. (**D**) *In situ* hybridization of Alu-sequences, revealing the presence of human cells (stained dark brown) throughout the tumor tissue, with the exception of the matrix and the endothelium, which is murine-derived (not-stained, light blue) in PDX4 tumor, ×20; (**E**) PDX4 tumor, ×50. (**F**) Negative immunohistochemistry for surface markers on lymphocytes in a PDX tumor, **1**) human CD45 (hCD45), **2**) hCD3, **3**) hCD4, **4**) hCD20; ×20.

**Supplementary Figure S6.** Neutrophil morphology. (**A**) Schematic representation of the typical morphology of murine hypersegmented neutrophils (modified from Pillay, J., Tak, T., Kamp, V.M., Koenderman, L. Immune suppression by neutrophils and granulocytic myeloid-derived suppressor cells: similarities and differences. *Cell Mol Life Sci* **70**, 3813–3827, doi:10.1007/s00018-013-1286-4 (2013). (**B**) Liquid cytology (*ThinPrep,* Cytyc Corporation; Boxborough, MA, USA) of the exudate from a tumor treated with anti-PD-1 in our study, showing neutrophils with multilobed and hypersegmented nuclei (magnification ×100), with one of them shown in more detail in (**C**) (magnification ×150). (**D**) Isolated peripheral neutrophil incubated in vitro with anti-PD-1 (50 µg/ml), whose nuclear morphology without staining is shown in an immunofluorescence detection image. (**E**) The multilobed, hypersegmented nucleus of the neutrophil previously shown in (**D**) is displayed here with a negative photo filter.

**Supplementary Figure S7.** Detection of neutrophils and nitrotyrosine in PDX tumors that received anti-PD-1 treatment and detection of anti-PD-1 in the original patient tumor tissue. (**A**) Representative images of the expression and localization of myeloperoxidase (red) and nitrotyrosine (green) by confocal microscopy in PDX4 tumors treated with anti-PD-1 monotherapy. Nuclei were stained with TO-PRO-3 (blue). Merged image corresponding to the necrotic areas reveals co-localization (yellow) of myeloperoxidase and nitrotyrosine, marked by white arrows. Scale bars, 30 µm. (**B**) Double immunofluorescence staining of myeloperoxidase and nitrotyrosine in PDX tumors. Representative merged images of the expression and localization of myeloperoxidase (red) and nitrotyrosine (green) by confocal microscopy in PDX tumors from control group, cisplatin-treated and anti-PD-1 and cisplatin (sequential) treated group. Nuclei were stained with TO-PRO-3 (blue). Co-localization (yellow) of myeloperoxidase and nitrotyrosine is marked by white arrows. Scale bars, 30 µm. (**C**) Detection of anti-PD-1 treatment antibody binding to infiltrated cells and necrotic areas of the original patient tumor tissue. Representative images of the location of anti-PD-1 bound (red) using confocal microscopy. Nuclei were stained with TO-PRO-3 (blue) and the images were merged. Scale bars, 100 µm.

**Supplementary Figure S8.** Anti-PD-1 antibody binding sites on neutrophil cell membrane surface. Representative confocal microscopy images of anti-PD-1 bound to PD-1 receptor (green color) on isolated murine neutrophils. The PD-1 active protein (in red) is bound to anti-PD-1 which is in turn is attached to the neutrophil through fragment crystallizable (Fc)-gamma receptors (FcγRs). Right panel shows merged images of co-localization (yellow color) of both receptors in the neutrophil. (**A**) Scale bars, 10 µm; (**B**) Scale bars, 20 µm; (**C**) Scale bars, 20 µm.
